# SARS-CoV-2 exploits steroidogenic machinery, triggers lipid metabolism for viral replication and induces immune response in Leydig cells of K18-hACE2 mice

**DOI:** 10.3389/fcimb.2025.1538461

**Published:** 2025-05-27

**Authors:** Salmo Azambuja de Oliveira, André Acácio Souza da Silva, Barry T. Hinton, Giovanni Freitas Gomes, Thiago Mattar Cunha, Paulo Sérgio Cerri, Estela Sasso-Cerri

**Affiliations:** 1 Department of Morphology and Genetics, Federal University of São Paulo, São Paulo, SP, Brazil; 2 Department of Cell Biology, School of Medicine, Virginia University, Charlottesville, VA, United States; 3 Center for Research in Inflammatory Diseases, Faculty of Medicine of Ribeirão Preto – USP, Ribeirão Preto, SP, Brazil; 4 Laboratory of Histology and Embryology, Department of Morphology, Genetics, Orthodontics and Pediatric Dentistry, School of Dentistry, São Paulo State University (UNESP), Araraquara, SP, Brazil

**Keywords:** COVID-19, testosterone, cholesterol, lipogenesis, nucleocapsid, cytokines, macrophages, electron microscopy

## Abstract

**Background:**

During COVID-19 pandemic, men had reduced serum testosterone and higher mortality rate than women. Variations in high density lipoprotein (HDL) levels were detected in severe COVID-19 individuals. We evaluated the response of testicular macrophages, steroidogenic activity and lipid metabolism of Leydig cells in SARS-CoV-2-infected K18-hACE2 mice.

**Methods:**

Testes were analyzed under light and electron microscope. Immunolocalization of human angiotensin converting enzyme (hACE2) and viral proteins (spike and nucleocapsid) were evaluated in association with the expression of viral recognition receptor*, Rigi*. Steroidogenesis was evaluated by the expression of *steroidogenic factor-1* (*Sf1*), and the immunolocalization of steroidogenic proteins and testosterone. Pro-inflammatory (TNF-α, IL-1β, IL-6), anti-inflammatory (IL-10) cytokines, macrophages (CD68 and CD163) and macrophage inhibitory factor (MIF) were detected by immunolocalization and Western blot. The expression of lipid metabolism genes (*Srebp, Dgat1 and Scarb1*) were investigated by RT-qPCR.

**Results:**

In the infected animals, the Leydig cells showed enhanced immunolocalization of hACE2, spike and nucleocapsid. The expression of *Rigi*, pro-inflammatory cytokines and number of macrophages increased, confirming viral infection. *Sf1* expression, steroidogenic proteins and testosterone were reduced whereas the expression of *Dgat1, Srebp and Scarb1* increased. Lipid droplets-enriched Leydig cells and viral particles in lipids were observed. The infected Leydig cells also showed enhanced pro-inflammatory cytokines immunolabeling.

**Conclusion:**

SARS-CoV-2 infects Leydig cells, activates its immune response and impairs steroidogenesis. The virus uses the steroidogenic machinery and induces lipid metabolism pathways for its survival and replication in these cells. These findings support the low testosterone and HDL levels in men with severe COVID-19.

## 1 Introduction

SARS-CoV-2, the virus responsible for severe acute respiratory syndrome (COVID-19), infects various organs through its interaction with angiotensin-converting enzyme 2 (ACE2), the main receptor of this virus (Hikmet et al., 2020). According to data from the Genotype-Tissue Expression Project, the testes exhibited the highest expression of ACE2 (Baughn et al., 2020), which was detected in the endothelium, peritubular myoid cells, spermatogonia, spermatocytes, spermatids, spermatozoa, Sertoli cells and Leydig cells (LCs) (Chen et al., 2020; Navarra et al., 2020; Wang and Xu, 2020; Fan et al., 2021; Ribeiro et al., 2023; Chen et al., 2023).

The testis is an immunologically privileged organ due to mechanisms that protect interstitial and germ cells against pathogens (Reviewed by Loveland et al., 2017). In the interstitial tissue, two distinct populations of macrophages were identified: 1) the recently recruited or transient CD68-positive macrophages (also called M1), characterized by the formation of reactive oxygen intermediates and the expression of pro-inflammatory cytokines (Wang et al., 2017; Zhu et al., 2022); 2) the resident population of CD163-positive macrophages (also called M2), which express anti-inflammatory cytokines, such as IL-10, and other factors (Hutson, 2006; Cutolo et al., 2022). These populations are regulated by macrophage inhibitory factor (MIF), which promotes the recruitment of macrophages and the functional polarization from M1 to M2 phenotype (Barbosa De Souza Rizzo et al., 2018; Pacheco-Fernández et al., 2019). In the testis, M2 macrophages are in close contact with LCs by cytoplasmic interdigitations (Hutson, 1992), establishing a crosstalk between these cells (Hutson, 2006). Studies have demonstrated that, under normal conditions, macrophages-derived cytokines play a role in the steroidogenic activity of LCs (Hutson, 2006; Reviewed by Loveland et al., 2017; de Oliveira et al., 2021).

During viral infections, the initial defense against RNA viruses comprises pattern recognition receptors (PRRs), which trigger innate immune responses (Reviewed by Rehwinkel and Gack, 2020). Among the PRRs, the cytoplasmic receptors RIG-I (*retinoic acid-inducible 1*) and MDA5 (*melanoma differentiation-associated 5*) play pivotal roles in the host’s antiviral response (Kato et al., 2006; Thoresen et al., 2021), confirming the innate immune activation following viral infection (Chibssa et al., 2021). The binding of RIG-I and MDA5 to viral RNA fragments induces their interaction with mitochondrial antiviral signaling protein (MAVS) (Thorne et al., 2021). This protein activates IKK-α/β/NF‐κB and IRF3/7 (*Interferon Regulatory Factor 3 and 7*) pathways, which induce the expression of cytokines and type I IFN genes, respectively (Zou et al., 2021).

During the Covid-19 pandemic, men were more vulnerable to the disease and had higher risk of developing severe forms of COVID-19 than women (Xie et al., 2020; Grasselli et al., 2020; Richardson et al., 2020). According to *in vitro* (Giannakopoulos et al., 2023; Chen et al., 2023) and *in vivo* (Li et al., 2022; Chen et al., 2023) studies, analysis of *post-mortem* testes from COVID-19 patients (Achua et al., 2021; Duarte-Neto et al., 2022; Costa et al., 2023) and literature reviews (Nassau et al., 2022; Ly et al., 2023; Dai et al., 2023). SARS-CoV-2 infects testicular cells, impairs the testicular structure and changes the testosterone levels, confirming the susceptibility of testes to SARS-CoV-2 infection in both patients and transgenic K18-hACE2 animal models (Giannakopoulos et al., 2023; Chen et al., 2023). Different single-stranded RNA viruses, such as hepatitis C virus (Sagan et al., 2006), picornavirus (Dorobantu et al., 2015) and Zika virus (Stoyanova et al., 2023) have shown that these pathogens exploit lipid metabolism, remodeling cellular pathways to create viral replication platforms. SARS-CoV-2, a single-stranded RNA virus, remodels the host cell for its replication through the formation of replication organelles (ROs), which consist of double-membrane vesicles (DMVs) (Cortese et al., 2020; Soares et al., 2023). Moreover, SARS-CoV-2 can reprogram the host cell lipid metabolism, increasing and accumulating lipid droplets (LDs) in the cell cytoplasm. These LDs are used as a viral replication platform and viral assembly (Dias et al., 2020; Soares et al., 2023).

The biosynthesis of testosterone by LCs is a complex steroidogenic process that depends on continuous cholesterol esters supply from LDs (Reviewed by Shen et al., 2016). In LCs, LDs are scattered throughout the cytoplasm or associated with mitochondria or surrounded by concentric layers of smooth endoplasmic reticulum (SER) cisternae, named spirally arranged cisternae (SAC) (Ohata, 1979; Mori et al., 1982). In LCs, LDs may be formed via SCAP/SREBP1 complex signaling, which induces: 1) endocytosis of high-density lipoprotein (HDL) through LDL receptor (LDLR) and HDL receptor (named scavenger receptor class B type I (SR-B1)), respectively (Shimizu-Albergine et al., 2016; Casado et al., 2021); and 2) lipogenesis via activation of diacylglycerol O-acyltransferase 1 (DGAT-1), a transmembrane enzyme located in SER that catalyzes the conversion of diacylglycerol and fatty acyl CoA to triacylglycerol, giving rise to LDs (Reviewed by Yen et al., 2008; Oresti et al., 2013). Thus, the initial step of steroidogenesis depends on the cholesterol transport via steroidogenic acute regulatory protein (StAR)-related lipid transfer protein 4 (StARD4) to the mitochondrial membrane (Reviewed by Miller, 2007). The cholesterol is internalized by StAR into the mitochondria, where the initial steps of steroidogenesis take place (Reviewed by Miller, 2007). Finally, in SER, androstenedione is converted into testosterone by 17β-HSD (Beattie et al., 2015; Wang et al., 2017). The critical regulator of steroidogenesis is the steroidogenic factor-1 (SF-1), a transcription factor and key regulator of genes that encode the steroidogenic enzymes (Sugawara et al., 1996; Manna et al., 2003). The steroidogenic process is susceptible to imbalance caused by different viral pathogens (Wu et al., 2016; Ma et al., 2016; Uraki et al., 2017), which can trigger an inflammatory process through the increase of cytokines. Under normal conditions, LCs are able to produce cytokines, such as TNF-α, IL-6, and IL-1β (Warren et al., 1990; Cudicini et al., 1997; Verhoeven et al., 1988; Gerendai et al., 2005; Semedo et al., 2022), which regulate steroidogenesis (Reviewed by Loveland et al., 2017; de Oliveira et al., 2021). However, under inflammatory conditions, high levels of cytokines impair LCs steroidogenesis and induce changes in the testis (Lysiak, 2004; Gualdoni et al., 2021).

To evaluate the severe acute respiratory syndrome (SARS), caused by the novel coronavirus (SARS-CoV-1) during the 2003 epidemic, McCray et al. (2007) developed a transgenic mouse (K18-hACE2) that expresses the human angiotensin-converting enzyme 2 (hACE2) and allows either SARS-CoV-1 (McCray et al., 2007) or SARS-CoV-2 infection (Moreau et al., 2020). Thus, this model has been used for the evaluation of antiviral therapeutic agents, vaccines and for the comprehension of COVID-19 pathogenesis (Bao et al., 2020; Rodrigues et al., 2022; Chen et al., 2023).

In the present study, we used SARS-CoV-2-infected K18-hACE2 transgenic mice to evaluate the immune response of testicular interstitial cells. To understand the cellular mechanisms involved in the virus-induced steroidogenic failure, the localization of the virus in the LCs and its relationship with steroidogenesis and lipid metabolism were investigated.

## 2 Materials and methods

### 2.1 Preparation of SARS-CoV-2 samples

The B1 strain of SARS-CoV-2 (Brazil/SPBR-02/2020 strain) was isolated from COVID-19 positive-tested patients at the Hospital of Ribeirão Preto of the University of São Paulo. The virus was propagated and titrated in Vero E6 cells under BSL-3 conditions at FMRP/USP (Ribeirão Preto, Brazil). Cells were cultured in DMEM medium supplemented with 10% fetal bovine serum and antibiotic/antimycotic drugs (Penicillin 10,000 U/mL; Streptomycin10,000 μg/mL). The viral inoculum was added to Vero cells in DMEM incubated at 37°C with 5% CO_2_ for 48 h, and the cytopathogenic effect was observed under microscope. Cell monolayer was collected, and the supernatant was stored at -70°C. Virus titration was made by the plaque-forming units (PFU).

### 2.2 K18-hACE2 transgenic mice: treatment and viral inoculation

Male mice (*Mus musculus*) of the C57BL/6 lineage (background), genetically modified (K18-hACE2), were utilized in this study. These mice have been used as model for SARS-CoV-2-induced disease since the infected animal presents clinical signs, and biochemical and histopathological changes compatible with the human disease (Bao et al., 2020; Rodrigues et al., 2022; Chen et al., 2023).

The animals were maintained and treated according to ARRIVE guidelines 2.0, and the protocol regarding the treatment of animals was approved by the Ethical Committee for Animal Research of Dental School, UNESP, Araraquara, São Paulo, Brazil (CEUA 021/2021).

Twenty K18-hACE2 mice, aged 12 weeks, were sourced from the Jackson Laboratory and bred at the Animal Special Breeding Center at the FMRP/USP. These animals were kept in the vivarium of the Center for Research in Virology II under 12h light and 12h dark cycle at controlled temperature (23 ± 2 °C) and humidity (65-75%), with water and food *ad libitum*. In a Biosafety Levels 3 laboratory (BSL3) at the FMRP/USP, the animals were divided into 2 groups: control group (CG; n=10) and infected group (IG; n=10). The animals of IG were inoculated with 5x10^4^ PFU of SARS-CoV-2 (in 40 μL) by intranasal route while the control mice were inoculated with DMEM. The animal’s weight and clinical signs were evaluated daily. It is important to emphasize that, according to previous studies (Moreau et al., 2020), the 5-day period of infection is the maximum period supported by the animal without suffering.

### 2.3 Histological procedures

After the fifth day of infection, the animals were anesthetized with 80 mg/kg BW of ketamine hydrochloride (Francotar, Virbac do Brasil Ind. Com. Ltda, Jurubatuba, Brazil, Reg.MA: 7.885) and 8 mg/kg BW of xylazine hydrochloride (Virbaxyl; Virbac do Brasil Ind. Com. Jurubatuba, Brazil, Reg.MA:7.899), and fragments of the left testes were collected and frozen at -80°C for molecular analysis or fixed in Karnovsky’s solution (as described below) for analysis under the transmission electron microscope (TEM). The right testes were fixed for 48 hours in 4% formaldehyde solution (Merck ERK, Germany) buffered with 0.1M sodium phosphate (pH 7.4), dehydrated in increasing concentrations of ethanol and embedded in historesin or paraffin. The sections were stained with H.E. for morphological and morphometric analyses. The paraffin sections were also adhered to silanized slides and submitted to immunohistochemistry and immunofluorescence analyses as described below.

### 2.4 Transmission electron microscopy

The samples were fixed for 16 hours in a solution containing 4% formaldehyde (Merck ERK, Germany) and 5% glutaraldehyde buffered with 0.1 M sodium cacodylate (pH 7.2) (Sasso-Cerri and Cerri, 2008). After washing in 0.1M sodium cacodylate (pH 7.2), the specimens were transferred to a 1% osmium tetroxide solution buffered with 0.1 M sodium cacodylate at pH 7.2 for 1 hour. After washings with distilled water, the specimens were immersed in 2% aqueous uranyl acetate for 1.5 hours, dehydrated in increasing concentrations of ethanol, treated with propylene oxide and then embedded in Araldite resin.

The Araldite polymerization was performed in an incubator oven at 56°C for 72 hours, and the semithin sections were obtained from an ultramicrotome (Ultracut UCT, Leica), and stained with 1% toluidine blue to select the area of ​​interest. The blocks were trimmed, and the ultrathin sections were collected on 200–300 mesh copper grids. The ultrathin sections were contrasted with an alcoholic solution of 2% uranyl acetate for 15 minutes and lead citrate. The ultrathin sections were examined in a transmission electron microscope FEI (TECNAI G2 Spirit, FEI Company) of the Biosciences Institute of UNESP-Botucatu (São Paulo, Brazil).

### 2.5 Nuclear area of Leydig cells

Six animals per group were used, and the analyses were performed using a DP-71 camera (Olympus, Tokyo, Japan) attached to an Olympus BX-51 microscope (Tokyo, Japan).

In non-serial HE-stained testicular sections, twenty fields of interstitial tissue per animal were randomly selected. In each field, the minor and major diameters of five LC nuclei were measured, totaling 100 LCs/animal under 400x. Typical LC nuclei showing ovoid shape and punctate peripherical chromatin were measured using an image analysis system (Image Pro-Express 6.0, Olympus). The nuclear area was calculated using the formula for obtaining the area of an ellipse: *A*=πab (a=length of the semi-major axis; b=length of the semi-minor axis).

### 2.6 Immunohistochemistry and immunofluorescence analyses

CD68 (ED1 macrophage marker) and CD163 (ED2 macrophage marker) were detected by immunohistochemistry. StAR (steroidogenic acute regulatory protein), 17β-HSD (steroidogenic enzyme), testosterone, IL-1β, TNF-α and IL-6 (pro-inflammatory cytokines), hACE2 (human angiotensin-converting enzyme 2), spike and nucleocapsid proteins (viral proteins) were detected by immunofluorescence.

Sections were immersed in 0.001 M citrate buffer (pH 6.0) and heated at 95°C in a microwave oven for 30 min for antigen recovery. For detection of CD68 and CD163 by immunohistochemistry, the sections were previously immersed in 2% hydrogen peroxide for endogenous peroxidase inactivation. All sections were incubated in 2% BSA for 30 minutes, and incubated at 4°C overnight with the following primary antibodies (**Table 1**): anti-CD68 polyclonal antibody; anti-CD163 monoclonal antibody; anti-StAR polyclonal antibody; anti-17β-HSD6 polyclonal antibody; anti-testosterone polyclonal antibody; anti-IL-1β polyclonal antibody, anti-IL-6 polyclonal IgG, anti-TNF-α monoclonal [52B83] antibody, anti-human ACE2 monoclonal antibody, anti-SARS-CoV-2 Spike Protein S1 and anti-SARS-CoV-2 nucleocapsid protein antibody. Sections incubated with anti-CD68 and anti-CD163 antibodies (**Table 1**) were washed in PBS and incubated at room temperature with biotinylated anti-mouse or anti-rabbit IgG secondary antibody and peroxidase-labeled-streptavidin (Universal Dako LSAB Kit, Dako Inc., Carpinteria, CA, USA). The sections were stained with 3.3’-diaminobenzidine (DAB: Dako Liquid DAB+Substrate Chromogen system, Dako Inc., Carpinteria, CA, USA), counterstained with Carazzi’s hematoxylin and mounted with Permount^®^ resin mounting medium. The testicular sections subjected to immunofluorescence reaction were washed in PBS and incubated in the dark with the following secondary antibodies (**Table 1**): Alexa Fluor^®^594 anti-rabbit antibody, Alexa Fluor^®^488 anti-mouse IgG antibody and Alexa Fluor^®^641 anti-donkey polyclonal antibody for 1 hour at room temperature. After washing in PBS, nuclear staining was performed with DAPI (Molecular Probes by Life Technologies; Carlsbad, CA, USA) for 5 min in the dark at room temperature, and the slides were mounted with Fluoromount^®^ mounting medium (Dako Inc., Carpinteria, CA, USA) or Fluro-Gell III Mounted Medium^®^ (Electron Microscopy Sciences). To check for possible unspecific binding of the secondary antibodies to the tissues, negative controls were performed by incubating sections with non-immune serum instead of primary antibodies.

**Table 1:** Primary and secondary antibodies.

Antibody name	Dilution	Catalog number	Manufacturer	RRID
Mouse anti-ACE2 monoclonal antibody	1:50	sc-73668	Santa Cruz Biotechnology, USA	AB_2861379
Rabbit anti-SARS-CoV-2 Spike Protein S1 Recombinant monoclonal antibody	1:250	MA5-36247	Invitrogen by Thermo Fisher Scientific	AB_2890589
Rabbit anti-SARS-CoV-2 nucleocapsid protein monoclonal antibody	1:3000	ab271180	Abcam, Cambridge, UK	–
Rabbit anti-IL-1β polyclonal antibody	1:400	ab9722	Abcam, Cambridge, UK	AB_308765
Goat anti-IL-6 (M19) polyclonal antibody	1:400	sc-1265	Santa Cruz Biotechnology, USA	AB_2127470
Mouse anti-TNF-α monoclonal [52B83] antibody	1:200	ab1793	Abcam, Cambridge, UK	AB_302615
Rabbit anti-CD163 monoclonal antibody	1:100	ab182422	Abcam, Cambridge, UK	AB_2753196
Rabbit anti-CD68 polyclonal antibody	1:100	ab125212	Abcam, Cambridge, UK	AB_10975465
Rabbit anti-17β-HSD6 polyclonal antibody	1:500	sc-393936	Santa Cruz Biotechnology, USA	AB_2891064
Rabbit anti-StAR polyclonal antibody	1:1000	PAS-95765	Invitrogen by Thermo Fisher Scientific	–
Mouse anti-testosterone polyclonal antibody	1:100	ab217912	Abcam, Cambridge, UK	AB_308765
Rabbit anti-IL-10 polyclonal antibody	1:500	sc-8438	Santa Cruz Biotechnology, USA	AB_627793
Rabbit anti-MIF polyclonal antibody	1:200	251415	ABBIOTEC, USA	AB_10636927
Rabbit anti-β-tubulin monoclonal antibody	1:8000	ab108342	Abcam, Cambridge, UK	AB_10866289
HRP-conjugated anti-rabbit secondary antibody	1:9000	A9169	Sigma-Aldrich, USA	
Alexa Fluor^®^488 anti-mouse antibody	1:1000	ab150113	Abcam, Cambridge, UK	AB_2576208
Alexa Fluor^®^488 anti-rabbit antibody	1:1000	ab150077	Abcam, Cambridge, UK	AB_2630356
Alexa Fluor^®^594 anti-mouse antibody	1:1000	ab150116	Abcam, Cambridge, UK	AB_2650601
Alexa Fluor^®^ 647 anti-rabbit antibody	1:1000	ab150075	Abcam, Cambridge, UK	AB_2752244
Alexa Fluor^®^647 anti-goat antibody	1:1000	ab150135	Abcam, Cambridge, UK	AB_2687955

#### 2.6.1 Number of CD68^+^ and CD163^+^ macrophages

In four non-serial testicular sections per animal (n=6), 20 fields of interstitial tissue were randomly captured under an Olympus BX-51 microscope (Olympus, Tokyo, Japan) equipped with a camera DP-71 (Olympus, Tokyo, Japan), at 400x. In these fields, a standardized area of ​​interstitial tissue per animal was measured using Image-Pro Express^®^ software (Olympus, Tokyo, Japan). In this area, the number of CD68- and CD163-positive macrophages was quantified, and the number of CD68 and CD163 macrophages per µm^2^ of interstitial tissue was calculated.

#### 2.6.2 Analysis of the immunofluorescent area

Immunofluorescent areas were analyzed using DFC 550 Camera (Leica, Germany) attached to a BM4000 B LED microscope (Leica, Germany) and the Leica Application Suite software (LAS 4.3, Leica, Germany). The StAR, 17β-HSD, testosterone, IL-1β, TNF-α and IL-6 immunofluorescent areas were measured at 400x. All parameters of the software, including exposure, gain and saturation as well as the threshold adjustment and color range were rigorously standardized for each immunolabeling analyzed so that only areas with intense red or green fluorescence were measured.

In four non-serial testicular sections per animal (n=6), the immunofluorescent area of each marker was measured in a total standardized area of interstitial tissue per animal, and the respective areas/mm² of interstitial tissue were calculated.

### 2.7 Double immunofluorescence analysis

To confirm the presence of SARS-Cov-2 in the testicular cells and hACE2 by immunolocalization, a double immunofluorescence staining was performed to detect hACE2 and spike in the same section. Moreover, to confirm if the LCs express an inflammatory profile, double immunofluorescences for detection of 17β-HSD6+IL-6, 17β-HSD6+IL-1β and StAR+TNF-α were also performed. The double immunofluorescence reactions were performed according to de Santi et al. (2022). After antigen recovery, the sections were incubated overnight at 4°C with the following primary antibodies (**Table 1**): anti-human ACE2 monoclonal antibody, anti-17β-HSD6 polyclonal antibody or anti-StAR polyclonal antibody. The day after, the sections were washed and incubated with Alexa Fluor^®^488 anti-mouse antibody or Alexa Fluor^®^594 anti-rabbit IgG antibody (**Table 1**) for 1 hour at room temperature. After washing in PBS, the sections were incubated overnight at 4°C with the following antibodies (**Table 1**): anti-SARS-CoV-2 Spike Protein S1, anti-IL-6 polyclonal antibody, anti-IL-1β polyclonal antibody and anti-TNF-α monoclonal antibody. The day after (third day), the sections were washed in high salt PBS and incubated in Alexa Fluor^®^594 anti-rabbit IgG antibody, Alexa Fluor^®^641 anti-donkey polyclonal antibody or Alexa Fluor^®^488 anti-mouse antibody (**Table 1**) for 1 hour at room temperature. After washing in PBS, nuclear staining was performed with DAPI for 5min in the dark at the room temperature, and the slides were mounted with Fluoromount^®^ mounting medium. Negative controls were performed following the same protocol and steps, except that the primary antibodies were replaced by non-immune serum. Immunofluorescence was analyzed using DFC 550 Camera (Leica, Germany) attached to a BM4000 B LED microscope (Leica, Germany) and the Leica Application Suite software (LAS 4.3, Leica, Germany). The immunofluorescence labeling in green (17β-HSD), red (IL-6) and yellow (resulted from the overlay of green and red fluorescence) were quantified. All software parameters, including exposure, gain, saturation as well as threshold and color range adjustments were rigorously standardized for each immunolabeling analyzed so that only areas with intense yellow fluorescence were computed. In the non-serial testicular sections, the immunofluorescent area for each marker was measured in a standardized total area of interstitial tissue per animal, and the immunofluorescent areas of 17β-HSD, IL-6 and 17β-HSD+IL-6/mm² of interstitial tissue were calculated for each animal.

### 2.8 Western Blot

Frozen testes samples (n=4) were homogenized with lysis buffer (50 mM Tris pH 8.0, 150 mM NaCl, 1 mM EDTA, 10% glycerol, 1% Triton X-100, 1 mM phenylmethylsulfonyl fluoride (PMSF) and 5 ng/mL of each protease inhibitor: Pepstatin, Leupeptin, Aprotinin **Table 2**, Antipain and Chymostatin (Sigma-Aldrich, code: P834-1ML, lot: #014M4024V)) and maintained overnight at 4°C. After centrifugation for 20 min at 8,944g, the supernatant was collected and the measurement of protein concentration was performed by Bradford (Sigma-Aldrich, EUA) assay. Protein samples (30µg) were separated in 12% or 20% SDS-PAGE and transferred to a nitrocellulose membrane 0.2µm (Bio-Rad Laboratories, USA). The membranes were treated for 1h with blocking solution containing 5% non-fat dry milk diluted in PBS/T (PBS/0.2% Tween 20) for nonspecific blocking and incubated overnight at 4°C with the following primary antibodies (**Table 1**): anti-MIF polyclonal IgG antibody and anti-IL-10 polyclonal IgG antibody diluted in blocking solution. After washes in 6 M urea buffer and 0.2% PBS/T, the membranes were incubated with HRP-conjugated anti-rabbit secondary antibody, diluted in blocking solution for 1h at room temperature. The reactions were detected using enhanced chemiluminescence system (ECL Plus, Boster Bio, USA).

**Table 2:** Sequence of primers used in qPCR.

Gene	NCBI access no:	Length (bp)	Oligonucleotides sequences (5’-3’)	Tm
*Human Ace2*	NM_001371415.1	21	F: GCTAGTCGACAGTGGGGAAAC	60,0°
*(Exxtend, Brazil)*		21	R: TGTCCTTGCCCTTATATAGTTCC	56,0°
*Mouse Ace2*	NM_001130513.1	20	F: GGACTAAGCCATGCAGGAAG	57,0°
*(Exxtend, Brazil)*		22	R: TGCAAAGAAAGGAGTCATTCAC	54,0°
*Rigi*	NM_172689.3	22	F: GGCAAGGGAACTGAAAACCATC	58,0°
*(Exxtend, Brazil)*		20	R: AAGCCGCACTTTCTGGTAGA	55,0°
*Dgat-1*	NM_010046.4	21	F: GAGGGGAAGACACAGAAGCTC	60,0°
*(Exxtend, Brazil)*		21	R: GTGTGTGCGCTCTCTCTCAAA	58,0°
*Scarb1*	NM_016741.2	23	F: CAGCCTGACAAGTCGCATGGCTC	63,0°
*(Exxtend, Brazil)*		22	R: AAAAGCACGCTGGCCCATGGTG	61,0°
*Srepb1*	Horie et al., 2013	20	F: TAGAGCATATCCCCCAGGTG	57,0°
*(Exxtend, Brazil)*		20	R: GGTACGGGCCACAAGAAGTA	57,0°
*Sf1/Nr5a1*	NM_001316687.1	20	F: TCTTGTCCTCCCCACAACCTG	58,0°
*(Exxtend, Brazil)*		20	R: GAGAGGTTCGCTGTGCTAGG	60,0°
*β-Actin*	NM_007393.5	18	F: CTGCGCTTCCTTTGTCCC	57,0°
*(Exxtend, Brazil)*		20	R: GACAATTGAGAAAGGGCGTG	55,0°

As positive controls, the membranes were stripped and re-probed with anti-β-tubulin monoclonal antibody (**Table 1**). The assays were performed in triplicate for CG and IG.

All bands were identified in Ponceau-stained membranes. The optical density (OD) of the band intensities of each lane (total protein) and the OD of the proteins of interest were quantified using Image Lab software (version 5.2.1, Bio-Rad). The results were obtained according to the normalized data using GraphPad Prism^®^ 8.3.4 software (GraphPad Software, CA, USA).

### 2.9 Reverse transcription and real-time polymerase chain reaction

The primers design was performed using the murine sequences available at the University of California, Santa Cruz (UCSC) Genome Browser and the Primer3 program (Untergasser et al., 2012) (**Table 2**).

Testis fragments (n=4) were collected and immersed in RNA Keeper stabilizing reagent (LGC Biotecnologia, Cotia, Brazil; 14-0002-01) and stored at -80°C. Testis RNA was isolated and purified using Aurum Total RNA Mini Kit (Bio-Rad Laboratories, Hercules, CA, USA; 732-6820). The cDNA was obtained using a High-Capacity cDNA Reverse Transcription Kit (Applied Biosystems, Cheshire, UK; 4368814) according to the manufacturer’s protocol. The real-time PCR was performed using the QuantStudio 3 Real-Time PCR instrument (Applied Biosystems, ThermoFisher; Life Technologies Holdings) and the PowerUp SYBR Green Master Mix (Applied Biosystems, Cheshire, UK; A25742). The qPCR cycling conditions were as follows: 40 cycles of denaturation at 95 °C for 15 s, annealing and extension at 60 °C for 1 min, and a final extension step with ramp rate 0,15°C/second at 95 °C for 1 5s. For gene expression analysis, the results were reported as mean ± SD, using the formula ΔCt = [Ct target gene–Ct housekeeping gene *β-actin*]. Relative expression is derived from log(2^–ΔΔCt), where ΔΔCt = ΔCt testes of IG – mean of ΔCt control group.

### 2.10 Statistical analysis

For the morphometric and immunofluorescence/immunohistochemistry analysis, 6 mice per group were used. For the Western blot and qPCR, 4 animals per group were used, and the experiments were performed in triplicate and duplicate, respectively. The data were checked for normal distribution by the Kolmogorov and Smirnov’s normality test and, according to the data distribution. Statistical comparisons were performed using *Student’s t test*, assuming significance at p ≤ 0.05, using the GraphPad Prism^®^ 4.3 software (GraphPad Software, CA, USA). The results were expressed as box and whiskers plots.

## 3 Results

### 3.1 Detection of hACE2 and spike protein (S1) in the testicular cells of the K18-hACE2 transgenic mice

**Figure 1:**
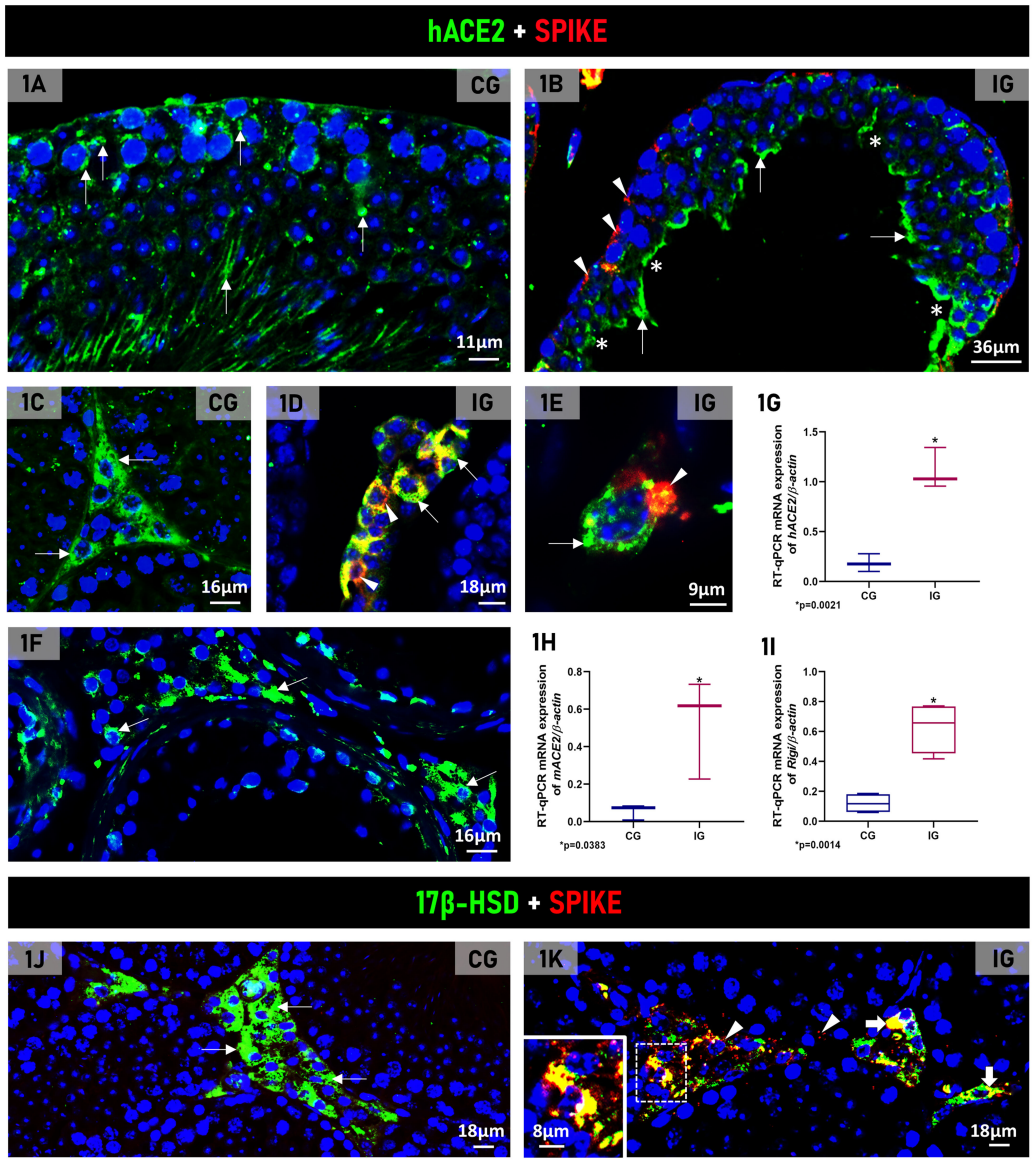
**(A–E)** Photomicrographs of testicular sections showing double immunofluorescence for detection of hACE2 (green color) and spike (red color) in animals from CG **(A, C)** and IG **(B, D, E)**. Nuclear staining with DAPI. In **(A, B)**, sections of seminiferous tubules show hACE2 immunolabeling (arrows) in both groups whereas spike immunolabeling (arrowheads) is observed only in IG **(B)**. Lack of germ cells in the epithelium (asterisks). In **(C-E)** hACE2 immunolabeling in the interstitial cells (arrows). In **(D)**, hACE2 (arrows), spike (arrowheads) and co-localization of hACE2+spike (yellow color) is observed in the interstitial cells. In **(E)**, a LC shows hACE2 (arrow) and spike (arrowhead). **(F)** Photomicrograph of human testicular section shows hACE2 immunolabeling in the Leydig cells (arrows) similarly to the transgenic mice **(C)**. **(G–I)** The mRNA expression of hACE2, mACE2 and Rigi is significantly greater in IG than in CG. **(J, K)** Photomicrographs of testicular sections showing double immunofluorescence for detection of 17b-HSD6 (green color) and spike (red color) in animals from CG and IG. Nuclear staining with DAPI. In **(J)**, LCs show only 17b-HSD immunolabeling (arrows) whereas in **(K)**, 17b-HSD7 and spike are co-localized in the LCs (yellow; thick arrows and inset). Spike immunolabeling is also observed in other interstitial cells, probably macrophages (red; arrowheads).

hACE2 was detected in seminiferous tubules and in the interstitial tissue in both groups (**Figures 1A–E**), whereas S1, as expected, was observed only in IG (**Figures 1B, D, E**). In this group, S1 immunofluorescence was observed in the epithelial cells (**Figure 1B**) and in the interstitial cells, including in the LCs (**Figures 1D, E**). In the human testis, evident hACE2 immunofluorescence was also observed in the Leydig cells (**Figure 1F**), similarly to the transgenic mice, confirming the reaction specificity. The *hAce2, mAce2* and *Rigi* mRNA expression increased 6-fold, 9-fold and 5-fold, respectively, (p=0.0021; p=0.0383; p=0.0014) in IG compared to CG (**Figures 1G–I**).

The double 17β-HSD and S1 immunofluorescence showed typical 17β-HSD immunofluorescence in the LCs of CG (**Figure 1J**) whereas, in IG, this double immunofluorescence confirmed the co-localization of S1 in the LCs (**Figure 1K**). S1 immunofluorescence was also observed in other interstitial cells, probably macrophages (**Figure 1K**).

### 3.2 SARS-CoV-2 activates macrophages and increases cytokines and MIF in interstitial tissue

**Figure 2:**
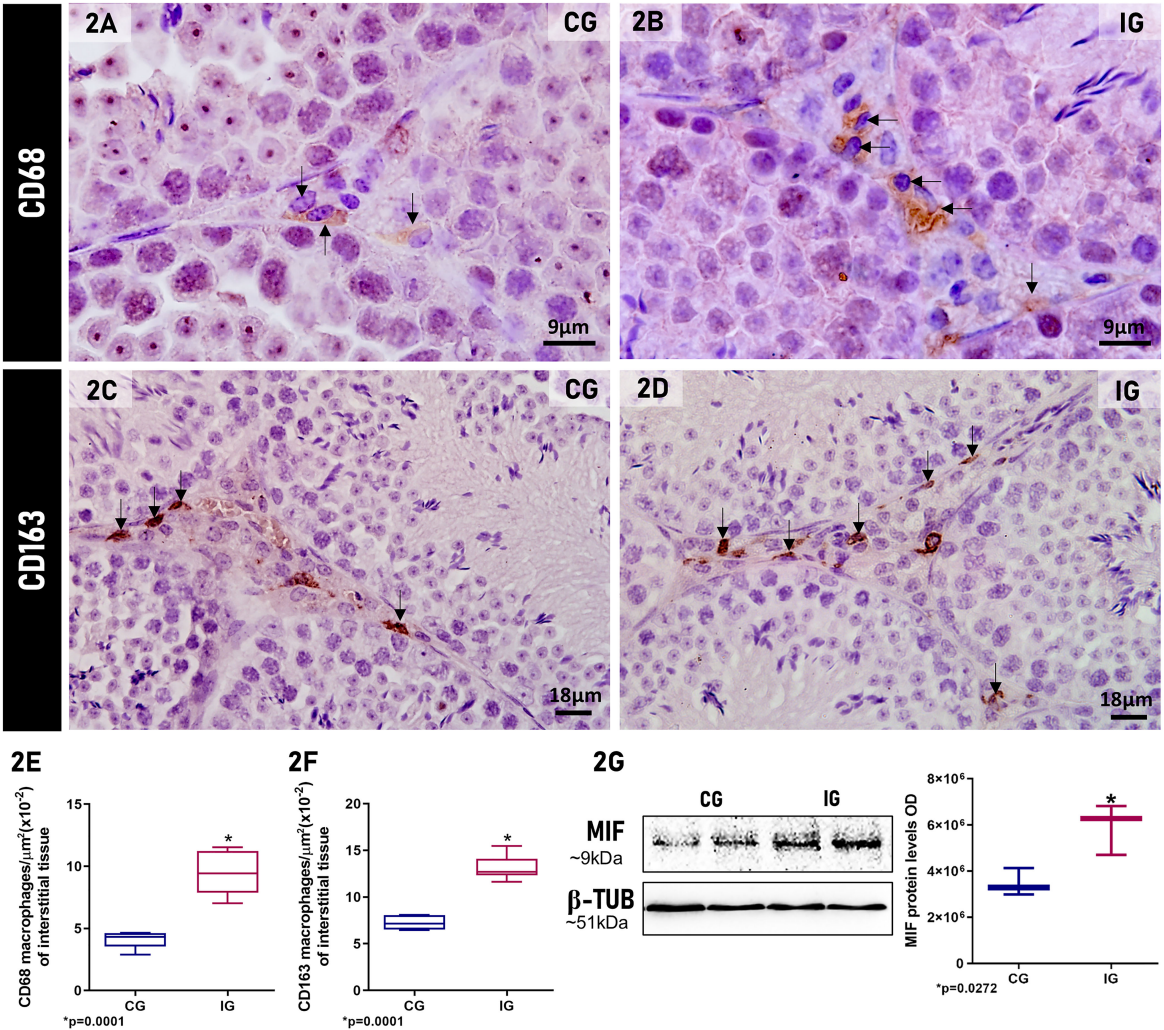
**(A–D)** Photomicrographs of testicular sections of animals from CG and IG submitted to CD68 **(A, B)** and CD163 **(C, D)** immunohistochemistry. Nuclear staining with hematoxylin. In **(A)** the interstitial tissue shows a few CD68^+^ macrophages (arrows) in comparison to the high incidence of these cells observed in IG **(B)**. In **(C, D)**, CD163-immunolabeled macrophages are seen (arrows); however, in **(D)**, note the high incidence of these macrophages (arrows) in comparison to CG. **(E, F)** The number of CD68 and CD163-immunolabeled macrophages per µm^2^ of interstitial tissue is higher in IG in comparison to CG. **(G)** Western blot analysis of MIF levels in testicular extracts shows strong bands at 9KDa in IG in comparison to CG. β-tubulin signal is observed in both groups. A significant increase in MIF levels optical density (OD) is observed in the animals from the IG when compared to CG. *p value.

In the interstitial tissue of testes of animals from the CG and IG, CD68 and CD163- immunolabeled macrophages (**Figures 2A–D**) were found in CG and IG. However, in IG, a high incidence of CD68^+^ and CD163^+^ macrophages (**Figures 2B, D**) were found compared to CG (**Figures 2A, C**). The quantitative analysis confirmed a significant increase (p=0.0001) in the number of CD68 and CD163- immunolabeled macrophages (2.3-fold and 1.7-fold, respectively) in IG compared to CG (**Figures 2E, F**). The analysis by Western blot showed a weak signal of MIF protein levels in CG; however, this factor increased 1.7-fold (p=0.0272) in IG (**Figure 2G**).

**Figure 3:**
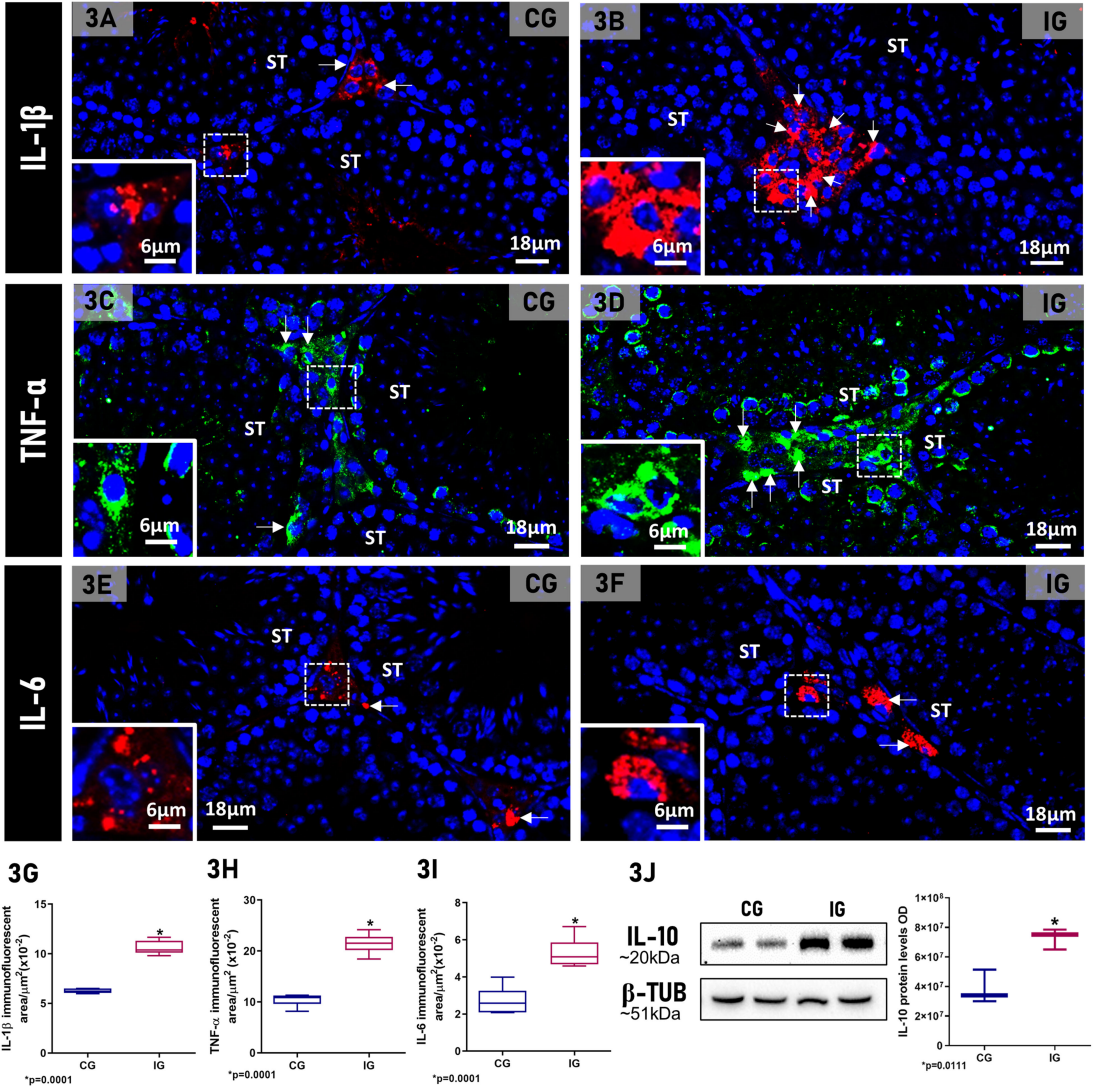
**(A–F)** Photomicrographs of testicular sections of animals showing IL-1β **(A, B)**, TNF-α **(C, D)** and IL-6 **(E, F)** immunofluorescence in the animals from CG and IG. Nuclear staining with DAPI. IL-1β, TNF-α and IL-6 immunoexpression is observed in the interstitial cells (arrows and insets) in both groups; however, in IG **(B, D, F)**, the immunoexpression of these cytokines is more intense and fills the cytoplasm of the interstitial cells (arrows and insets). Seminiferous tubules (ST). **(G–I)** IL-1β, TNF-α, and IL-6-immunofluorescent areas increased significantly in IG when compared to CG. **(J)** IL-10 weak signal at 20KDa is observed in CG when compared to a strong signal in IG. β-tubulin signal is observed in both groups. A significant increase of IL-10 optical density (OD) is observed in IG when compared to CG. *p value.

In testicular sections from both CG and IG groups, IL-1β, TNF-α and IL-6 immunoexpression were detected in the interstitial cells (**Figures 3A–F**). However, an intense immunoexpression for these cytokines was observed in the interstitial cells of the IG animals (**Figures 3B, D, F**) when compared to CG (**Figures 3A, C, E**). The immunofluorescent area of IL-1β, TNF-α and IL-6 increased 1.7-fold, 2-fold and 1.9-fold, respectively, (p=0.0001) in IG compared to CG (**Figures 3G–I**). The analysis by Western blot showed a weak signal of IL-10 protein levels in CG in comparison to 1.8-fold increase (p=0.0111) in the levels of this protein in IG (**Figure 3J**).

### 3.3 Infection by SARS-CoV-2 impairs LCs structure and steroidogenesis

**Figure 4:**
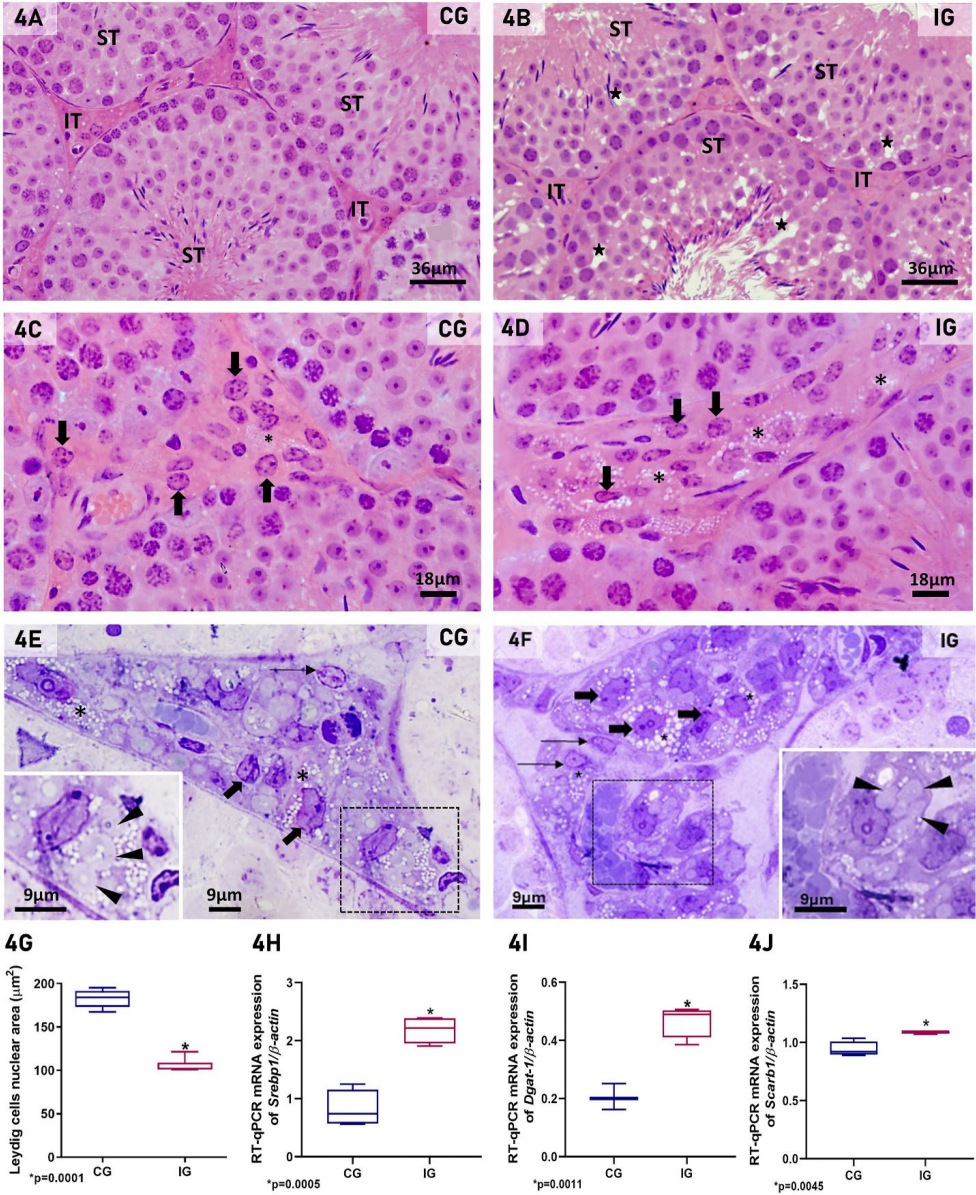
**(A–F)** Photomicrographs of testicular sections of animals from CG and IG stained with H.E. **(A-D)**, and semithin sections stained with toluidine blue **(E, F)**. In **(A)**, normal histoarchitecture of the seminiferous tubules (ST) and interstitial tissue (IT) is observed in CG. However, in **(B)** (IG), intraepithelial spaces are seen in the seminiferous tubules (ST; stars). IT, interstitial tissue. In **(C)**, the interstitial tissue exhibits typical LCs with round/ovoid nucleus (thick arrows) whereas in **(D)**, these cells show irregular and reduced nucleus (thick arrows), and numerous lipid inclusions (asterisks) in comparison to CG. In **(E, F)**, semithin sections show interstitial tissue containing macrophages (thin arrows) and LCs (thick arrows). Numerous and large lipid inclusions (asterisks) are observed in the LCs of IG when compared to CG. Note the spirally arranged cisternae in these cells (insets; arrowheads). **(G)** Significant reduction of LC nuclear area is observed in IG in comparison to CG. **(H–J)** A significant increase in the mRNA expression of *Srebp1, Dgat-1 and Scarb1* is observed in IG when compared to CG. *p value.

In H.E.-stained testicular sections of CG, the seminiferous tubules and interstitial tissue showed organized epithelial histoarchitecture and typical interstitial cells, respectively (**Figure 4A**). However, in IG, the seminiferous tubules showed numerous intraepithelial spaces (**Figure 4B**).

Either in CG or IG, typical steroidogenic organelles were found in the Leydig cells, including lipid inclusions and the smooth endoplasmic reticulum forming spirally arranged cisternae (SAC). In both H.E.-stained sections and semithin sections, the CG exhibited typical LCs with round/ovoid nucleus (**Figure 4C**), lipid droplets and SAC (**Figures 4C, E**). However, in IG, these cells showed irregular and reduced nucleus (**Figure 4D**), numerous lipid droplets filling the cytoplasm (**Figures 4D, F**) and the SAC were apparently smaller than in CG (**Figures 4E, F**). The nuclear area of the LCs was significantly reduced in IG animals (p=0.0001) when compared to CG (**Figure 4G**). The analysis by RT-qPCR showed that *Srebp1, Dgat-1* and *Scarb1* gene expression increased 2.6-fold, 2.2-fold and 1.2-fold, respectively, in IG compared to CG (**Figures 4H–J**).

**Figure 5:**
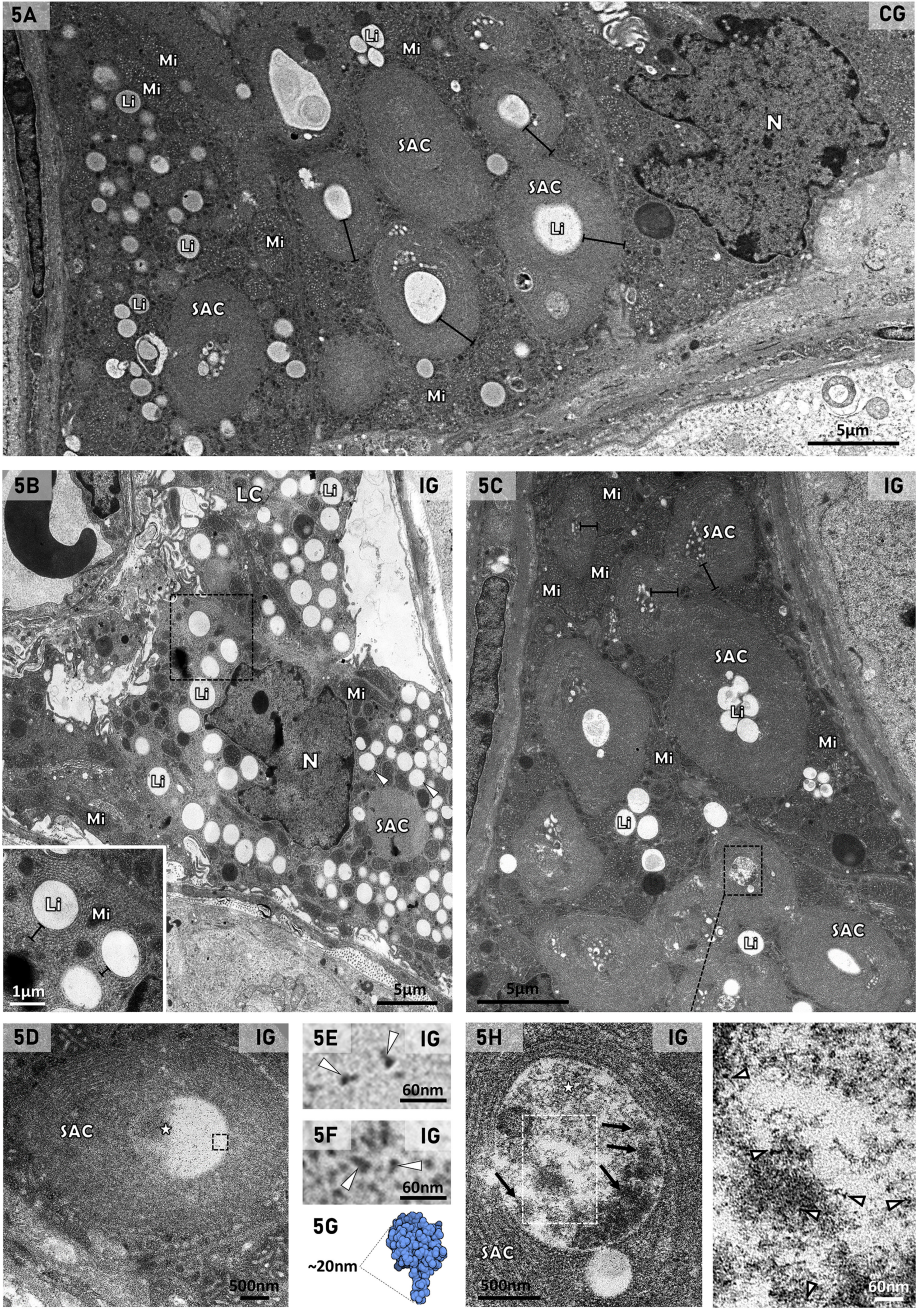
**(A–H)** Electron micrographs of LCs in the testis of animals from the CG **(A)** and IG **(B-I)**. In **(A-C)**, the cytoplasm of LCs shows mitochondria (Mi), lipid inclusion (Li) and spirally arranged cisternae (SAC) delimiting lipid inclusions. Note that in **(B)**, numerous lipid droplets (Li) are filling the cytoplasm, and some of them are apparently larger than in **(A)**. In **(B)** (inset) and **(C)**, the layers of some SAC are thinner (brackets) than in CG. N (nucleus). In **(D, H)**, the Li delimited by concentric SAC are filled by an electron opaque granular material (stars). In **(E, F)** (high magnification of **D**), the granular material shows small structures (arrowheads) similar to nucleocapsid proteins (as illustrated in **G**). In **(H)** (high magnification of **C**), the granular material forms spheroid structures measuring around ~140nm, and similar to viral particles (arrows). In the white box (inset), nucleocapsid protein-like structures are observed (arrowheads). In **(G)**, the crystal structure of SARS-CoV-2 nucleocapsid protein is illustrated. DOI: https://doi.org/10.2210/pdb6M3M/pdb.

**Figure 6:**
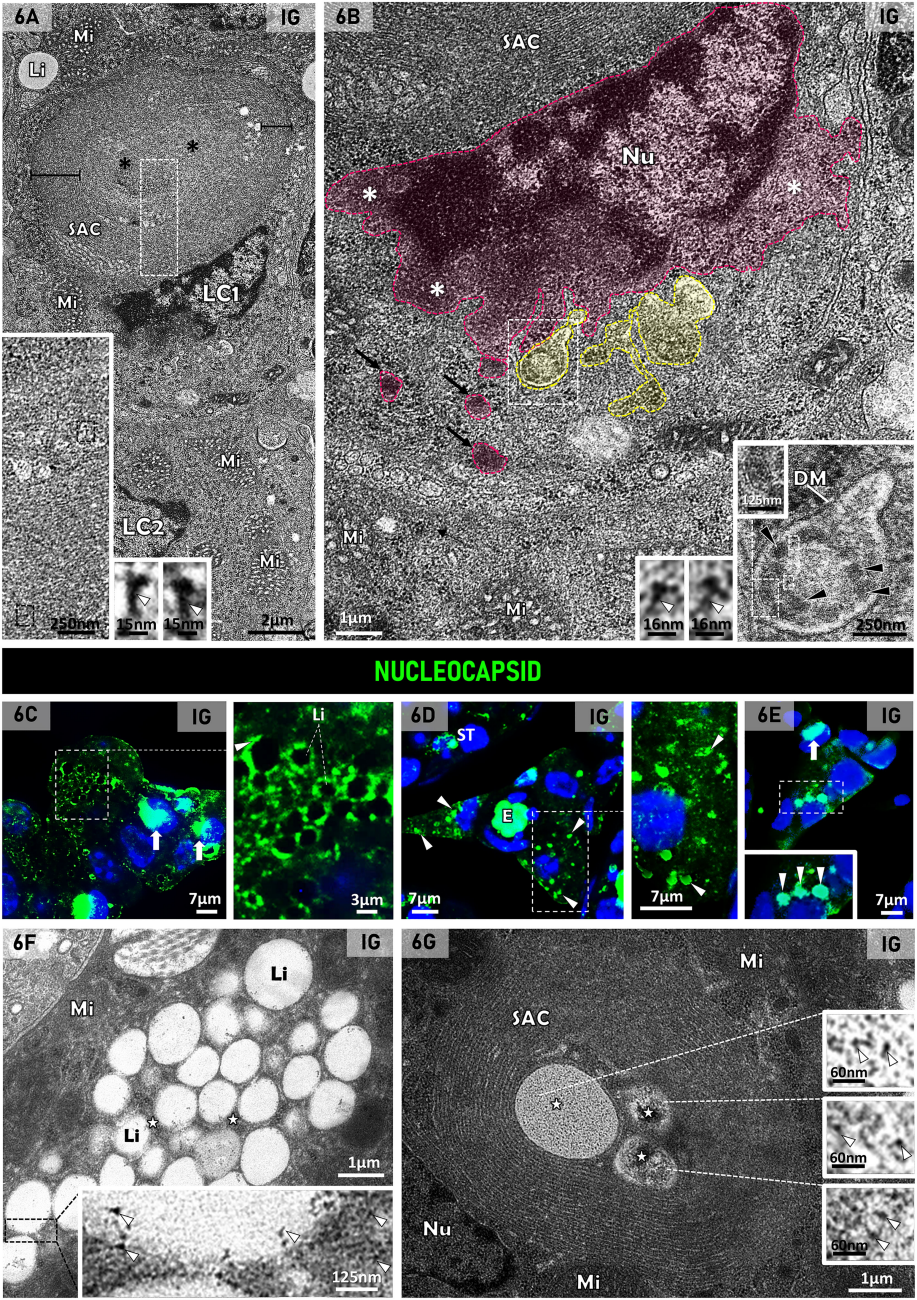
**(A, B)** Electron micrographs of Leydig cells of infected animal. In **(A)** the Leydig cells (LC) show mitochondria (Mi) and lipid inclusion (Li). In LC1, a large spirally arranged cisternae (SAC) is delimiting a central region containing a thin granular material (asterisks). Under high magnification (white box - inset), the granular material shows electron opaque structures similar to SARS-CoV-2 nucleocapsid proteins (black boxes and insets; white arrowheads). In **(B)**, high magnification of LC1 **(A)**, note the irregular dilations of the nuclear membrane (pink dotted line and asterisks). Some nuclear dilations are seen in cross section due to folding (black arrows). Double-membrane vesicles are also observed next to the nucleus (yellow dotted line). One of them (inset), showing evident double membrane (DM), contains enveloped viral particles measuring around 120nm and surrounded by a membrane bilayer (black arrowheads and inset). The white boxes under high magnification show nucleocapsid proteins (white arrowheads). Mitochondria (Mi); Nucleus (Nu). **(C–E)** Photomicrographs of interstitial tissue showing nucleocapsid proteins immunolabeling in the Leydig cells of infected animals. Nuclear staining with DAPI. In **(C)**, specific immunolabeling (white box and inset) is surrounding the lipid inclusion (Li) and within and/or in close contact with nuclei (white arrows). In **(D, E)**, Leydig cells show punctate immunofluorescent masses of nucleocapsid proteins in the cytoplasm (arrowheads and insets). Blood vessel with erythrocytes **(E)**. ST, seminiferous tubule. **(F, G)** Electron micrographs of Leydig cell regions in infected animals. Note nucleus (Nu), lipid inclusion (Li), mitochondria (Mi) and spirally arranged cisternae (SAC). Similarly to the immunolocalization shown in **(C-E)**, electron dense granular material (stars) is also observed in the Li periphery and in the core of SAC. Note that in these regions, nucleocapsid proteins are observed (insets; arrowheads).

Under TEM, the cytoplasm of the LCs showed mitochondria and SAC, delimiting lipid droplets in both CG and IG groups (**Figures 5A–C**). In IG, numerous lipid droplets were filling the cytoplasm; some of them were larger than those of CG (**Figures 5A–C**). The SAC were apparently smaller in IG than those of CG, and an electron opaque granular material was usually found in the central core of these whorls (**Figures 5D, H**). This granular material showed electron opaque structures similar to nucleocapsid (**Figures 5D–G**, **6A, F, G**), as well as spheroid structures measuring around ~140nm, similar to viral particles (**Figure 5H**).

In some interstitial regions of IG, LCs with atypical nucleus were found (**Figure 6A**). These cells showed irregular dilations of the nuclear membrane; due to folding, some of them were seen in cross section (**Figure 6B**). Double-membrane vesicles were also observed next to the nucleus. In some of them, enveloped viral particles measuring around 120nm were observed. In these viral particles, electron opaque structures similar to nucleocapsid were also found (**Figure 6B**).

In IG, nucleocapsid protein immunolabeling was observed surrounding the lipid droplets, within and/or in close contact with the nuclei and/or as immunofluorescent masses through the cytoplasm (**Figures 6C–E**). These findings were similar to those observed under TEM, which showed electron opaque granular material containing nucleocapsid proteins in the periphery of lipid droplets and in the core of SAC (**Figures 6F, G**).

**Figure 7:**
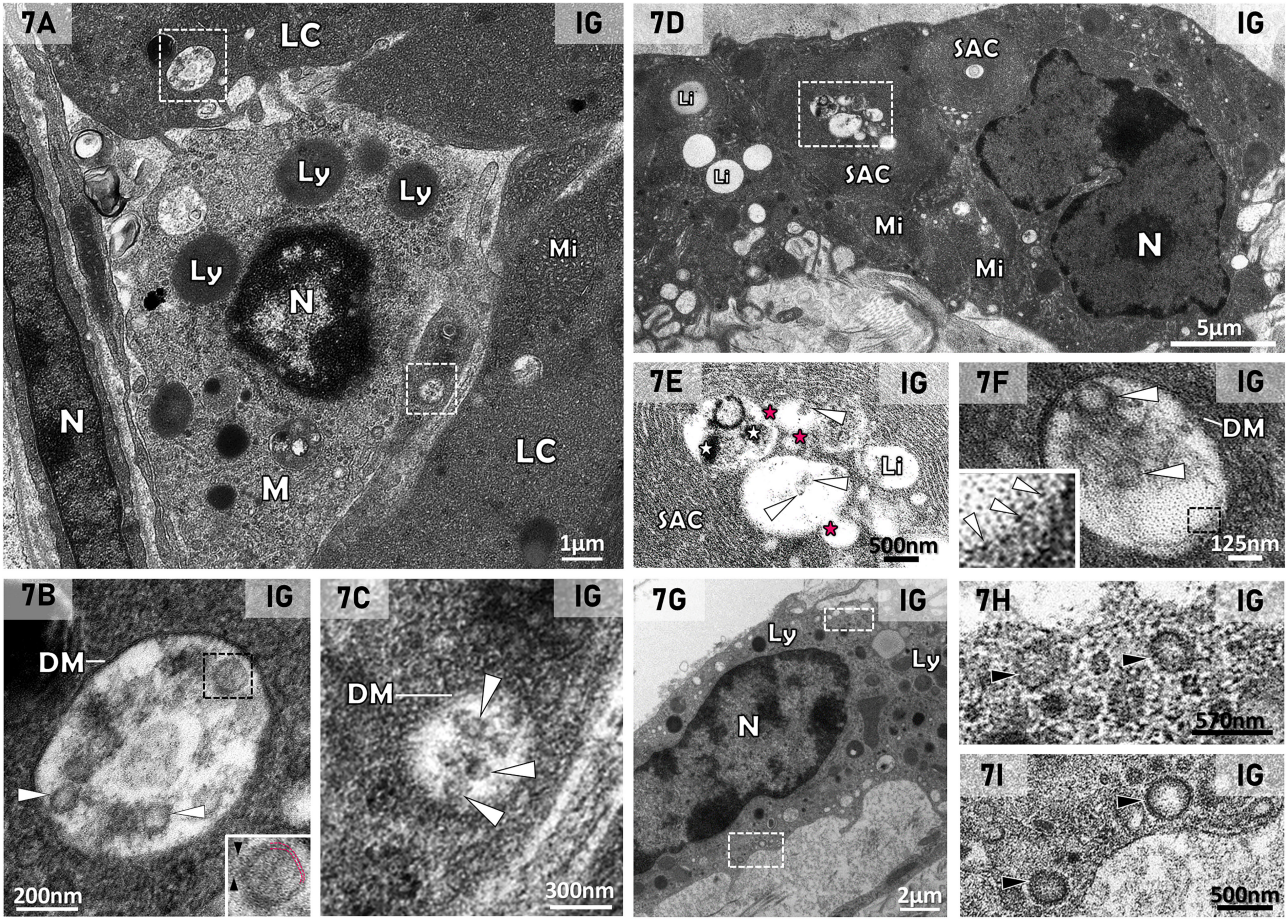
**(A–I)** Electron micrographs of interstitial tissue of testis of animal from the IG. In **(A)**, regions of Leydig cells (LC) with typical mitochondria (Mi) in close contact to a macrophage (M) containing several lysosomes (Ly). N, nucleus. In **(B)** (upper white box of **A**) and **(C)** (lower white box of **A**), vesicles showing typical double membrane (DM) and containing enveloped viral particles measuring around 130nm (arrowheads) are observed in the cytoplasm of LCs. In the black box of 7B (inset), an enveloped viral particle with membrane bilayer (pink dotted lines) and spike proteins (black arrowheads) are observed. In **(D)**, a Leydig cell shows mitochondria (Mi) and spirally arranged cisternae (SAC) surrounding lipid inclusion (Li). N, nucleus. In **(E)** (high magnification of white box of **D**), the SAC surrounds lipid inclusions, which are fusing with each other (pink asterisks). An electron dense material (white asterisks) and some enveloped viral particles-like structures measuring around 140nm (arrowheads) are observed. In **(F)**, a region of LC shows a large double membrane vesicle (DMV) containing enveloped viral particles measuring around 120nm (arrowheads). In **(G)**, a macrophage containing numerous lysosomes (Ly) and coated vesicles (white boxes) spread through the cytosol. In **(H, I)** (high magnification of **G**), coated vesicles are spread through the cytosol and morphologically different from the viral particles **(B, C, E, F)**.

LCs in close contact with macrophages containing lysosomes were also usually observed in IG (**Figure 7A**). In the cytoplasm of these LCs, small or large double membrane vesicles containing enveloped viral particles were found (**Figures 7B–D, F**). In the SAC core, small and large lipid inclusions were fusing with each other (**Figure 7E**). Electron opaque material and viral particles measuring around 100nm were also observed in these lipid droplets (**Figure 7E**). In the macrophages, coated vesicles (**Figures 7A, G–I**) were also found and could be differentiated from the viral particles (**Figures 7B, C, E, F**).

**Figure 8:**
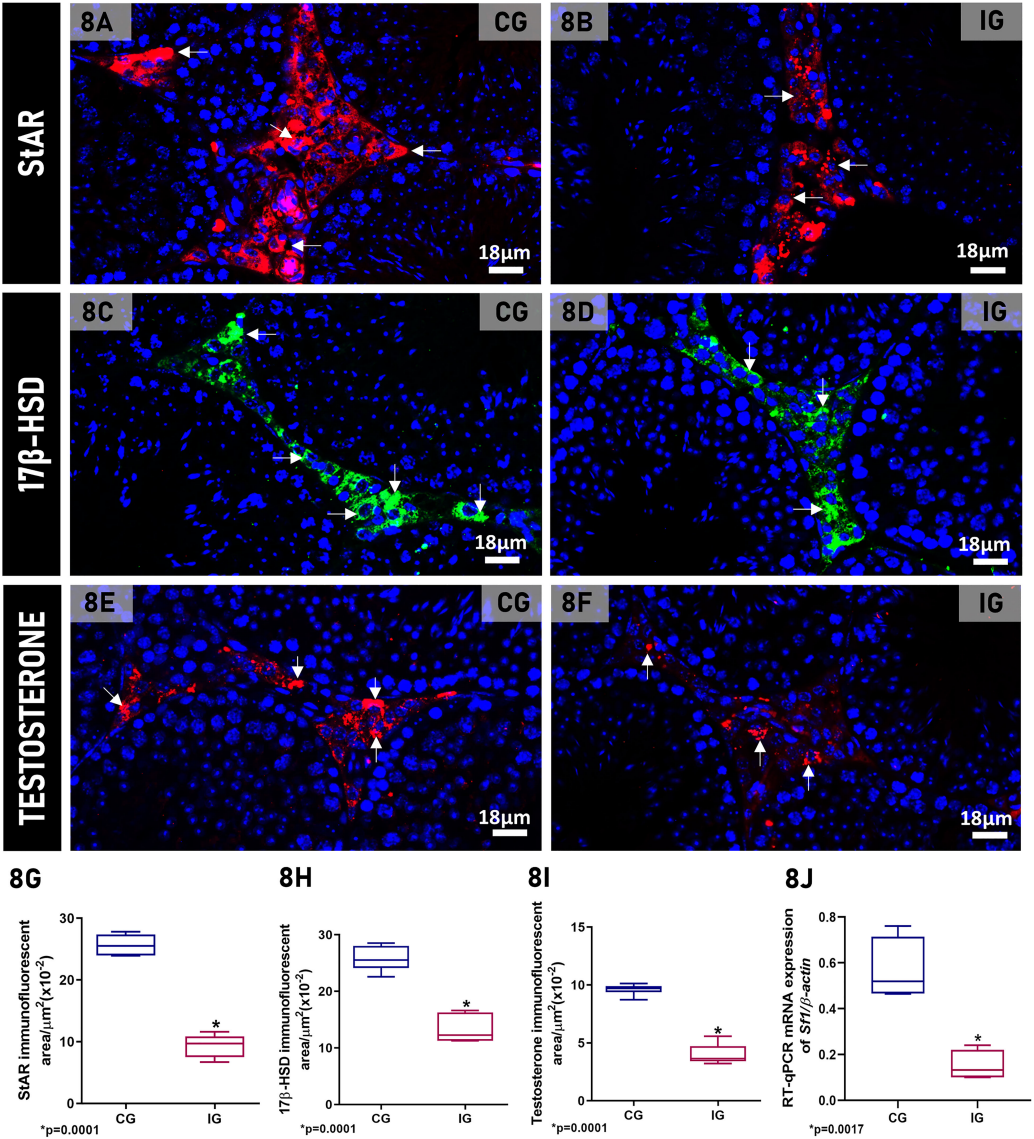
**(A–F)** Photomicrographs of testicular sections subjected to immunofluorescence for detection of StAR **(A, B)**, 17β-HSD **(C, D)** and testosterone **(E, F)**. Nuclear staining with DAPI. Immunofluorescent labeling for StAR, 17β-HSD and testosterone are observed in the cytoplasm of LCs (arrows) in both groups; however, note the weak immunoexpression in IG when compared to CG. **(G–I)** Significant reduction of StAR, 17β-HSD and testosterone immunofluorescent areas is observed in IG. **(J)** The mRNA expression of *Sf1* is significantly decreased in IG in comparison to CG.

In the testicular sections of animals from both CG and IG groups, StAR, 17β-HSD and testosterone immunoexpression was detected in the LCs cytoplasm. However, the immunoexpression of these markers was weaker in IG (**Figures 8B, D, F**) than in CG (**Figures 8A, C, E**). This finding was confirmed by the significant reduction (65%, 47% and 56%, respectively) of the immunofluorescent areas of StAR, 17β-HSD and testosterone (p=0.0001) in IG (**Figures 8G–I**). The steroidogenic factor-1 (*Sf1)* mRNA expression also reduced (74%; p=0.0017) in IG when compared to CG (**Figure 8J**).

### 3.4 Immunoexpression of TNF-α, IL-6 and IL-1β in infected LCs

**Figure 9:**
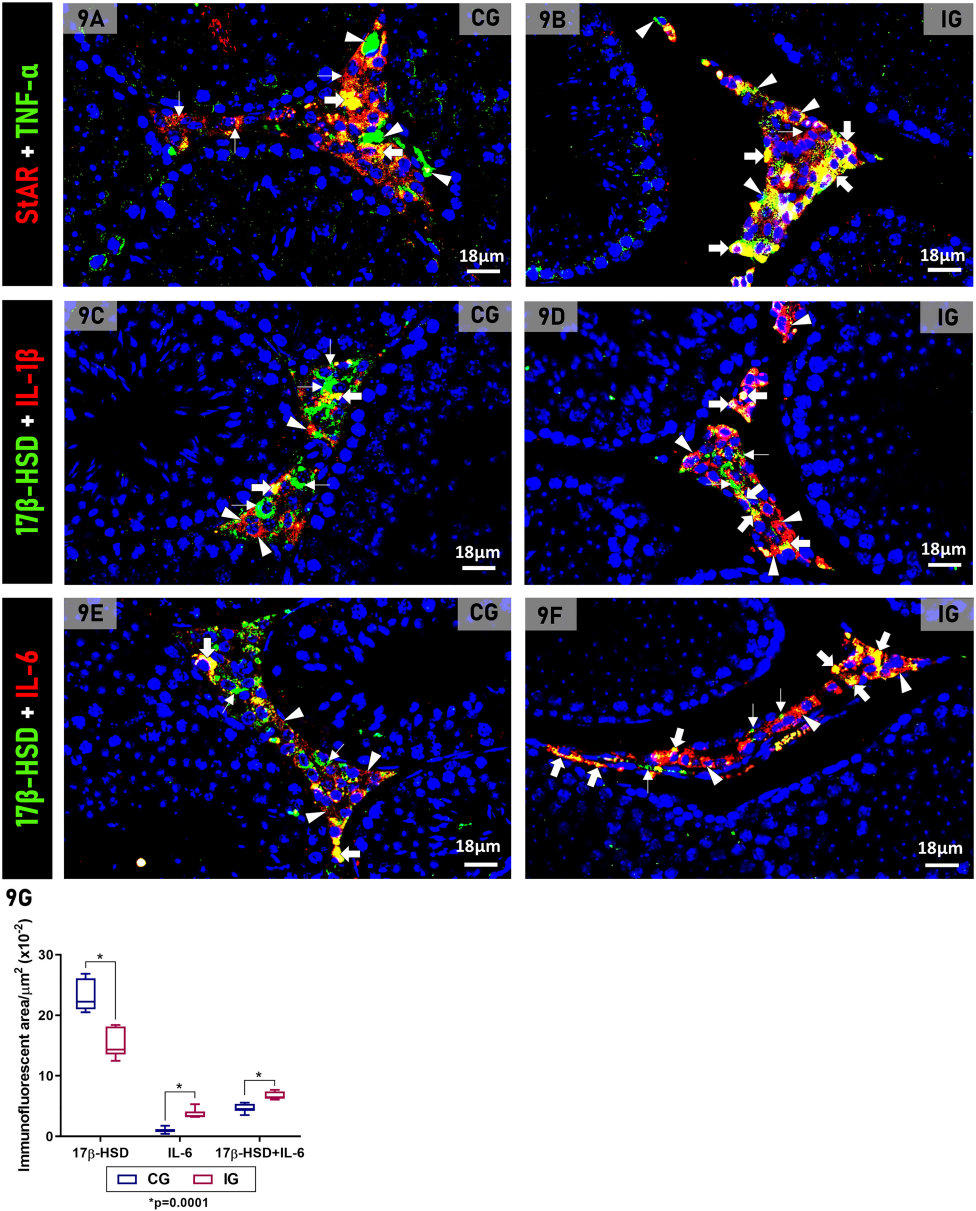
**(A–F)** Photomicrographs of testicular sections of animals showing double immunofluorescence for detection of StAR+TNF-α **(A, B)**, 17β-HSD+IL-1β **(C, D)** and 17β-HSD+IL-6 **(E, F)** in the animals from the CG and IG. TNF-α immunoexpression (green, arrowheads), IL-1β or IL-6 immunoexpression (red, arrowheads) is observed in macrophages and other interstitial cells. LCs show StAR (red, thin arrows) or 17β-HSD immunoexpression (green, thin arrows) as well as double TNF-α, IL-1β or IL-6 + 17β-HSD immunoexpression (yellow, thick arrows). In **(B, D, F)** note that TNF-α (green; arrowheads), IL-1β or IL-6 immunolabeled interstitial cells (red; arrowheads) and double immunolabeled LCs (yellow, thick arrows) are more evident than in CG **(A, C, D)**. **(G)** 17β-HSD, IL-6 and 17β-HSD+IL-6 immunofluorescent areas in CG and IG. *p value.

Either in CG or in IG, the interstitial tissue showed StAR or 17β-HSD-immunolabeled LCs, in green fluorescence, as well as TNF-α, IL-1β and IL-6 in other interstitial cells (macrophages), in red fluorescence. The co-localization of these cytokines with steroidogenic proteins (yellow fluorescence) was also detected in the LCs (**Figures 9A–F**). Notably, in IG, in parallel to the reduction of LCs markers (StAR and 17β-HSD; green fluorescence), an evident increase was noted in the cytokines-immunolabeled interstitial cells (in red) and in the co-localized StAR+TNF-α, 17βHSD+IL-1β and 17βHSD+IL-6 immunolabeling (yellow fluorescence), when compared with CG (**Figures 9B, D, F**). The measurement of the co-localized immunofluorescent areas confirmed that 17β-HSD+IL-6 immunolabeling increased 1.3-fold (p=0.0001) in IG compared to CG (**Figure 9G**).

## 4 Discussion

**Figure 10:**
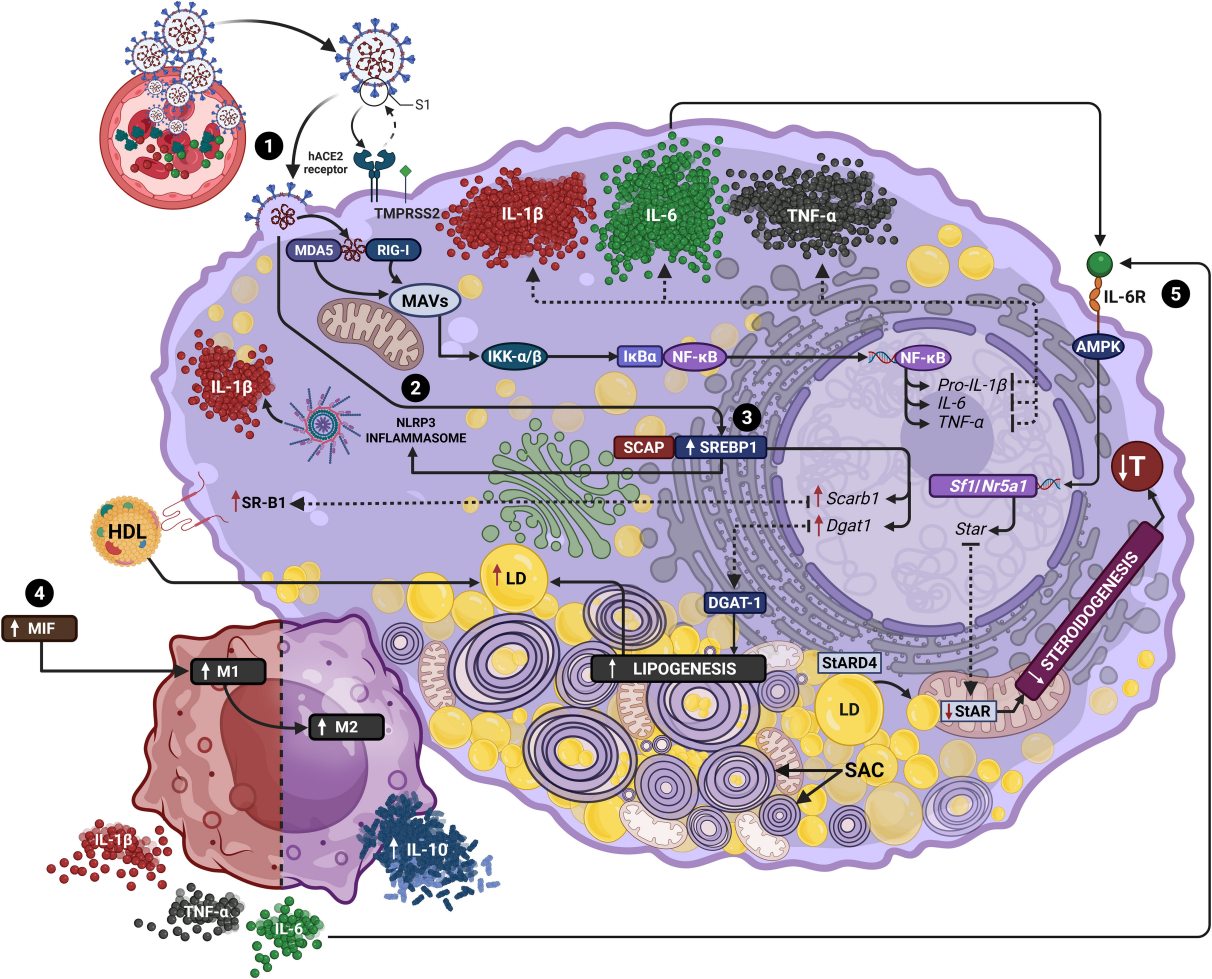
Schematic representation of Leydig cell following SARS-CoV-2 infection. 1) LC is infected through the binding of SARS-CoV-2 spike protein (S1) to the hACE2 receptor. Upon exposure to viral RNA, the cytoplasmic receptors RIG-I and MDA5 are activated, triggering MAVs and NF-kB pathways, inducing NF-kB translocation to the nucleus and production of pro-inflammatory cytokines, such as IL-1β, IL-6 and TNF-α. 2) The virus induces SCAP/SREBP-1 transcription factor, which induces NLRP3 inflammasome complex activation, leading to cytokines production, such as IL-1β. 3) SCAP/SREBP-1 also activates the expression of *Scarb1* and *Dgat1*, which induces the production of SR-B1 (HDL receptor) and lipogenesis in the spirally arranged cisternae (SAC), respectively. 4) High levels of MIF induce the recruitment of M1 macrophages and the production of cytokines. MIF also mediates the polarization of M1 to M2 macrophages, which produce IL-10. 5) High levels of IL-6, derived from macrophages and LC, bind to IL-6 receptor in the LC, dysregulate AMPK pathway and downregulate Sf1/Nr5a1 gene, impairing StAR and steroidogenic activity, culminating in low T levels.

In this study, the testes of the transgenic mice K18-hACE2 expressed human *Ace2 (hACE2)* and were infected by SARS-CoV-2. Although some studies have demonstrated SARS-CoV-2 virus particles in the LCs, the subcellular localization of the virus and the structural and functional changes of these cells in response to the viral infection have been poorly addressed. In the LCs of animals from the IG, assembled viral particles and/or nucleocapsid proteins were observed in close contact or within lipids droplets and spirally arranged cisternae, confirming a tropism of SARS-CoV-2 for LC steroidogenic machinery. Indeed, the infection induced a significant reduction of steroidogenesis and *Sf-1* downregulation (**Figure 10**). In contrast, the high concentration of lipids droplets in the infected LCs, in association with *Srebp1, Dgat1 and Scarb1* overexpression, indicated increased lipogenesis and cholesterol uptake (**Figure 10**). These findings confirm that SARS-CoV-2 exploits the LCs machinery and the lipid metabolism pathways in favor of its replication. The intense immunoexpression of cytokines in these hypofunctional steroidogenic cells suggests a transition of these cells to an immunological phenotype to eliminate the virus.

### 4.1 SARS-CoV-2 infects testicular cells

In addition to the testicular expression of *hACE2*, an enhanced immunolocalization of hACE2 was observed in the interstitial cells and seminiferous epithelium of the transgenic K18-hACE2 mice, corroborating previous findings (Chen et al., 2020; Navarra et al., 2020; Wang and Xu, 2020; Fan et al., 2021; Ribeiro et al., 2023; Chen et al., 2023). High levels of ACE2 have been demonstrated in human Leydig cells (Wang and Xu, 2020). This finding agrees with our results since enhanced hACE2 immunoexpression was observed in the Leydig cells of the non-infected transgenic mice, confirming that this cell type is susceptible to infection. It is important to emphasize that *mAce2* was also expressed in the testes of the transgenic mice, confirming that the expression of *hAce2* did not interfere in the expression of *mAce2.* In IG, either *hACE2* or *mACE2* expression increased significantly, confirming that the testicular tissues were infected by the virus. The increased expression of both viral recognition sensor (*Rigi)* and TNF-α corroborates this finding since studies have shown that ACE2 expression is upregulated by cytokines as well as by the activation of pathways triggered by viral sensors (Zhuang et al., 2020; da Silva et al., 2024).

Although spike and nucleocapsid proteins were detected in the testicular sections by immunofluorescence, the ultrastructural analyses under TEM were essential to confirm the presence of viral particles in the LCs as well as to better understand their association with the steroidogenic hypofunction. For the identification of the viral particles, we considered several major criteria, such as the presence of nucleocapsid proteins, which may be surrounded by an envelope in case of assembled virus, the size of assembled virus (60 to 150 nm) and the presence of double membrane vesicles (DMVs) (Revised by Hopfer et al., 2021; Revised by Bullock et al., 2021). Indeed, all these structures were found in the LCs of IG animals, including the presence of DMVs containing assembled viral particles measuring around 120nm. Similar findings were also observed in the LCs of *post-mortem* testes from COVID-19 patients (Achua et al., 2021; Duarte-Neto et al., 2022; Costa et al., 2023). In the present study, in addition to DMVs, we also observed nucleocapsid proteins in the lipid droplets, spirally arranged cisternae and in the nuclear membrane dilations of LCs under TEM. Most of these findings were confirmed by the immunolocalization of nucleocapsid protein, indicating that the virus seems to hijack the LCs and exploit their cellular structures for its replication.

### 4.2 SARS-CoV-2 activates testicular macrophages recruitment and the production of cytokines

According to Hales (2002), an increased number of macrophages in the testes is associated with testicular inflammation since macrophages produce high levels of cytokines and impair testicular function (Lysiak et al., 2003; Lysiak, 2004; Altavilla et al., 2012). Post-mortem testes of COVID-19 individuals showed increased number of CD68 macrophages (Costa et al., 2023; Duarte-Neto et al., 2022). The recruitment of M1 (CD68) macrophages and the functional polarization of these cells to M2 (CD163) macrophages are induced by MIF (Barbosa De Souza Rizzo et al., 2018; Pacheco-Fernández et al., 2019). In the present study, a significant increase in the number of M1 macrophages was also found in the infected animals; this finding is corroborated by the high MIF protein levels as well as by the intense immunoexpression of IL-1β, TNF-α, and IL-6, the main pro-inflammatory cytokines produced by M1 macrophages under inflammatory conditions (Cutolo et al., 2022). In addition to M1, a significant increase in the number of M2 macrophages was also observed in IG, suggesting a possible polarization of M1 to M2 macrophages, likely due to high MIF levels. Since M2 macrophages produce IL-10 under normal conditions (Huang et al., 2018), preventing a detrimental immune response against infections (Carlini et al., 2023), it is possible that the increased number of these cells and the high IL-10 levels, observed in IG, are related to the maintenance of testicular homeostasis, ensuring the pathogen elimination.

Studies have demonstrated that RIG-I activation induces an inflammatory response in infected testes (Zhu et al., 2013; Wu and Chen, 2014; Wu et al., 2016). Indeed, the significant increase in *Rigi* expression, in IG, corroborates SARS-CoV-2 infection in testicular cells as well as enhanced immunoexpression of IL-1β, TNF-α and IL-6 (**Figure 10**). Our data are supported by the findings of Giannakopoulos et al. (2023), which demonstrated overexpression of *IL-1β, TNF-α*, and *IL-6* mRNA levels in the testes of SARS-CoV-2-infected K18-hACE2 animals.

### 4.3 SARS-CoV-2 infection impairs LCs steroidogenic activity

Our results showed a considerable immunoexpression of cytokines (TNF-α, IL-6, and IL-1β) in the interstitial cells of control animals, corroborating previous studies which confirmed that these cytokines regulate steroidogenesis under physiological conditions (Reviewed by Loveland et al., 2017; de Oliveira et al., 2021). However, the viral infection impaired steroidogenic activity in the testes of the K18-hACE2 mice. *In vivo* and *in vitro* studies have demonstrated that high levels of cytokines impair the steroidogenic cascade, inhibiting the expression of key proteins such as StAR, 3β-HSD, and 17β-HSD/P450c17 (Hales, 1992; Leisegang and Henkel, 2018; Soliman et al., 2021). *In vitro* studies have shown that TNF-α impairs testosterone production by LCs and decreases mRNA levels of CYP11A1 and CYP17A1 (Xiong and Hales, 1993; Li et al., 1995). According to Samir et al. (2017), TNF-α and IL-6 inhibit androgen secretion through CYP17A1 cytochrome P450 and luteinizing hormone/choriogonadotropin receptor (LHCGR) mRNA downregulation. Moreover, TNF-α reduces the expression of INSL3 (*insulin like 3*), HSD3B1 (*3 beta- and steroid delta-isomerase 1*) and NOS3 (*Nitric Oxide Synthase 3*) while IL-6 reduces the expression of LHCGR and StAR (Samir et al., 2017). IL-6 also reduces the release of androgens due to *Sf-1* downregulation (McIlmoil et al., 2015; De Silva et al., 2018). Our findings showed enhanced TNF-α, IL-6, and IL-1β immunoexpressions in association with significant reduction in the immunoexpression of testosterone, StAR, and 17β-HSD along with the downregulation of *Sf1* gene, corroborating the anti-androgenic effect of these pro-inflammatory cytokines in the LCs (**Figure 10**). Several studies have demonstrated reduction in the testosterone levels of COVID-19 patients (Lanser et al., 2021; Salonia et al., 2021; Li et al., 2023). Moreover, in the testis of patients who died from COVID-19, the levels of StAR, 3β-HSD, and 17β-HSD were significantly reduced and associated with low intratesticular testosterone levels (Costa et al., 2023), corroborating our findings.

### 4.4 SARS-CoV-2 uses LC steroidogenic machinery and induces lipogenesis for its own replication

The interruption of steroidogenesis, observed in IG, was associated with a notable presence of lipid droplets in the cytoplasm of LCs. According to Hatano et al., 2016, the SF-1 deficiency causes lipid droplets accumulation in LCs due to suppression of the steroidogenic proteins StAR and CYP11A1. The treatment of mice with diethylcarbamazine also reduces testosterone and induces a high concentration of lipid droplets in the cytoplasm of Leydig cells (Saraiva et al., 2006, 2008). Therefore, the accumulation of lipid droplets is caused by blockade of steroidogenesis, including the mobilization of cholesterol, which is re-esterified and stored in lipid droplets (Saraiva et al., 2006). These data corroborate our findings, indicating that the lipid accumulation observed in the LCs of the animals from the IG may be, at least in part, due to impaired steroidogenic activity. On the other hand, *in vitro* studies have confirmed that coronavirus plays a role in lipid metabolism (Yan et al., 2019), and lipid droplets accumulation has been detected in SARS-CoV-2-infected Vero E6 cells and type II pneumocytes of lung tissues from COVID-19 patients (Nardacci et al., 2021). SARS-CoV-2 virus optimizes its replication through the formation of Replication organelles (ROS) named DMVs in infected cells (Nardacci et al., 2021), and approximately 40% of the ROs are in close contact to lipid droplets, whose fatty acids are derived from the smooth endoplasmic reticulum, a crucial organelle for the biogenesis of DMVs (Ricciardi et al. (2022). These processes depend on the production of phospholipids through the activation of several pathways and genes (Reviewed by Lagace and Ridgway, 2013), including lipogenic genes, such as *Dgat1* (Reviewed by Gao et al., 2019). In Calu-3 cells, lipid droplets are involved in the SARS-CoV-2 infectious cycle based on the concept that the nucleocapsid protein drives *Dgat-1* and *Dgat-2* expression, promoting lipid droplets formation (Yuan et al., 2021). Therefore, SARS-CoV-2 is able to reprogram lipid metabolism via *Dgat1*, leading to accumulation of lipid droplets, which have been used by the virus as a viral assembly platform (Dias et al., 2020).

Under TEM, the LCs of IG showed nucleocapsid-like proteins surrounding lipid droplets, and SAC were circumscribing a central region containing a thin granular material containing nucleocapsid-like proteins. Moreover, viral particles were found surrounding or within lipid droplets. To confirm if the infection induced lipogenesis, the expression of *Dgat1* was evaluated, and the overexpression of this gene reinforced the idea that lipid droplets are not only accumulated due to steroidogenic blockade but are also derived from “*de novo*” lipogenesis activated by the viral infection. Since lipogenesis depends on the cholesterol supply via endocytosis through the receptors LDLR and HDLR (Shimizu-Albergine et al., 2016; Casado et al., 2021), we evaluated the expression of *Srebp1 and Scarb1* genes, which encodes, respectively, the transcription factor SREBP (regulator of biosynthesis and uptake of cholesterol and fatty acids) and HDLR. Indeed, the testis of animals from the IG showed significant overexpression of *Srebp* and *Scarb1*. These findings corroborate previous studies, which have shown that SCAP/SREBP accelerates the accumulation of cholesterol in cells by increasing intracellular cholesterol biosynthesis (Lee et al., 2023; Soares et al., 2023), a crucial step for SARS-CoV-2 replication (Abu-Farha et al., 2020). Moreover, either *Srebp* knockdown or the inactivation of SREBP inhibits SARS-CoV-2 replication and lipid droplets formation in Calu-3 cells (Dias et al., 2020). Therefore, our findings show that SARS-CoV-2 exploits lipid metabolism pathways in the LCs through the activation of SCAP/SREBP-1, which triggers *Scarb1* and *Dgat1* overexpression, culminating in cholesterol endocytosis and lipogenesis (**Figure 10**).

It is important to emphasize that clinical studies have demonstrated that serum HDL levels reduce drastically, compared to LDL, in men with high risk of death by COVID-19 (Feingold, 2023; Al-Zadjali et al., 2024). Moreover, this pattern was more prevalent in men than in women (Parra et al., 2023). In this context, in addition to the consumption of cholesterol (HDL) by cells of diverse organs, such as hepatic cells as well as adrenocortical cells, which produce high levels of cortisol in severe Covid-19 patients (Tan et al., 2020), we can suggest that the vulnerability of male patients to Covid-19 may be related, at least in part, to the susceptibility of testes to infection due to: 1) the high levels of ACE2 in the testicular cells (Fan et al., 2021; Edenfield and Easley, 2022) and 2) the presence of lipid-rich LCs containing a well-developed steroidogenic machinery appropriate for the viral replication, a process that requires significant HDL uptake from the bloodstream.

### 4.5 Activation of LCs immune response following SARS-CoV-2 infection

The increased immunoexpression of cytokines in the steroidogenically hypofunctional LCs of IG is intriguing, and points to a transition of the steroidogenic profile of these cells to an immune profile triggered by the viral infection. During mumps virus (Wu et al., 2016) and Zika virus (Ma et al., 2016; Uraki et al., 2017) infections, both LCs and Sertoli cells trigger innate immune responses, leading to increased concentrations of inflammatory cytokines, including IL-1β, IL-2, IL-6, and TNF-α, in response to the recognition of viral RNA. This ability is associated with the expression of viral RNA recognition receptors, such as *Rigi* and *Mda5*, in these cells, allowing an immune response and a subsequent inflammatory process (Zhu et al., 2013, 2014; Wang et al., 2021). Therefore, the significant increase of *Rigi* expression associated with the intense immunoexpression of pro-inflammatory cytokines reinforces the idea that LCs may undergo a transition from a steroidogenic profile to an immunological profile, exerting an antiviral immune response (**Figure 10**). Recent studies demonstrated that increased SREBP induces NLRP3 inflammasome complex activation and production of IL-1β (Soares et al., 2023; Liu et al., 2024). Therefore, the LC lipid metabolism activation does not seem to be related solely to viral replication but may also be involved in the activation of the LCs immune response to the infection. Future studies are needed to elucidate the viral and cellular mechanisms involved in the LC immune response.

## 5 Conclusion

The expression of hACE2 and the presence of viral particles in the interstitial cells confirm the susceptibility of the testes to SARS-CoV-2 infection in the K18-hACE2 mice. The infection triggers a testicular immune response through the activation of M1 and M2 macrophages and cytokines production by both macrophages and LCs. The high levels of cytokines and/or viral infection impair steroidogenesis, contributing to enhanced concentration of lipids in LCs. The virus also exploits steroidogenic machinery for lipogenesis in favor of its replication, inducing cholesterol endocytosis and contributing to increased lipids in LCs. These findings support the low serum testosterone and cholesterol levels in men with high risk of death by COVID-19 and highlight possible therapeutic targets to prevent these effects.

## Data Availability

The original contributions presented in the study are included in the article. Further inquiries can be directed to the corresponding author.

## Glossary

17β-HSD: 17β-hydroxysteroid dehydrogenase
3β-HSD: 3β-Hydroxysteroid dehydrogenase
ACE2: Angiotensin-Converting Enzyme 2
BSL3: Biosafety Level 3 Laboratory
CARDs: Caspase Activation and Recruitment Domain
COVID-19: severe acute respiratory syndrome
CG: Control Group
CYP11A1: Cholesterol side-chain cleavage enzyme
DGAT-1: Enzyme Diacylglycerol O-Acyltransferase-1
DMVs: Double-Membrane Vesicles
EGFR: Epidermal Growth Factor Receptor
FBS: Fetal Bovine Serum
hACE2: human Angiotensin-Converting Enzyme 2
HDL: High Density Lipoprotein
IG: Infected Group
INSL3: Insulin Like 3
IRF3/7: Interferon Regulatory Factor 3 and 7
LCs: Leydig cells
LDL: Low Density Lipoprotein
LDs: Lipid Droplets
MDA5: Melanoma Differentiation-Associated Protein 5
MAVS: Mitochondrial Antiviral Signaling Protein
MIF: Macrophage Migration Inhibitory Factor
NLRP3: NLR family pyrin domain containing 3
NOS3: Nitric Oxide Synthase 3
PFU: Plaque-Forming Units
PMSF: Phenylmethylsulfonyl Fluoride
PRRs: Pattern Recognition Receptors
RIG-I: Retinoic Acid-Inducible 1
ROs: Replication Organelles
S1: Spike Protein
SAC: Spirally Arranged Cisternae
SCAP: Cleavage-Activating Protein
SCARB1: Scavenger Receptor Class B type 1
SER: Smooth Endoplasmic Reticulum
SF-1: Steroidogenic Factor-1
SREBP1: Sterol Regulatory Element Binding Transcription Factor 1
StAR: Steroidogenic Acute Regulatory Protein
StARD4: Lipid Transfer Protein 4
TEM: Transmission Electron Microscopy.
