# Correction: SARS-CoV-2 exploits steroidogenic machinery, triggers lipid metabolism for viral replication and induces immune response in Leydig cells of K18-hACE2 mice

**DOI:** 10.3389/fcimb.2025.1702430

**Published:** 2026-01-05

**Authors:** Salmo Azambuja de Oliveira, André Acácio Souza da Silva, Barry T. Hinton, Giovanni Freitas Gomes, Thiago Mattar Cunha, Paulo Sérgio Cerri, Estela Sasso-Cerri

**Affiliations:** 1 Department of Morphology and Genetics, Federal University of São Paulo, São Paulo, SP, Brazil; 2 Department of Cell Biology, School of Medicine, Virginia University, Charlottesville, VA, United States; 3 Center for Research in Inflammatory Diseases, Faculty of Medicine of Ribeirão Preto – USP, Ribeirão Preto, SP, Brazil; 4 Laboratory of Histology and Embryology, Department of Morphology, Genetics, Orthodontics and Pediatric Dentistry, School of Dentistry, São Paulo State University (UNESP), Araraquara, SP, Brazil

**Keywords:** COVID-19, testosterone, cholesterol, lipogenesis, nucleocapsid, cytokines, macrophages, electron microscopy

In the published article, **Figure 4F** was retouched to eliminate three artifacts from toluidine blue staining. These artifactual blue deposits were retouched for aesthetic purposes, and the retouching does not impact the interpretation of the data nor the conclusions of the study.

**Figure 4:**
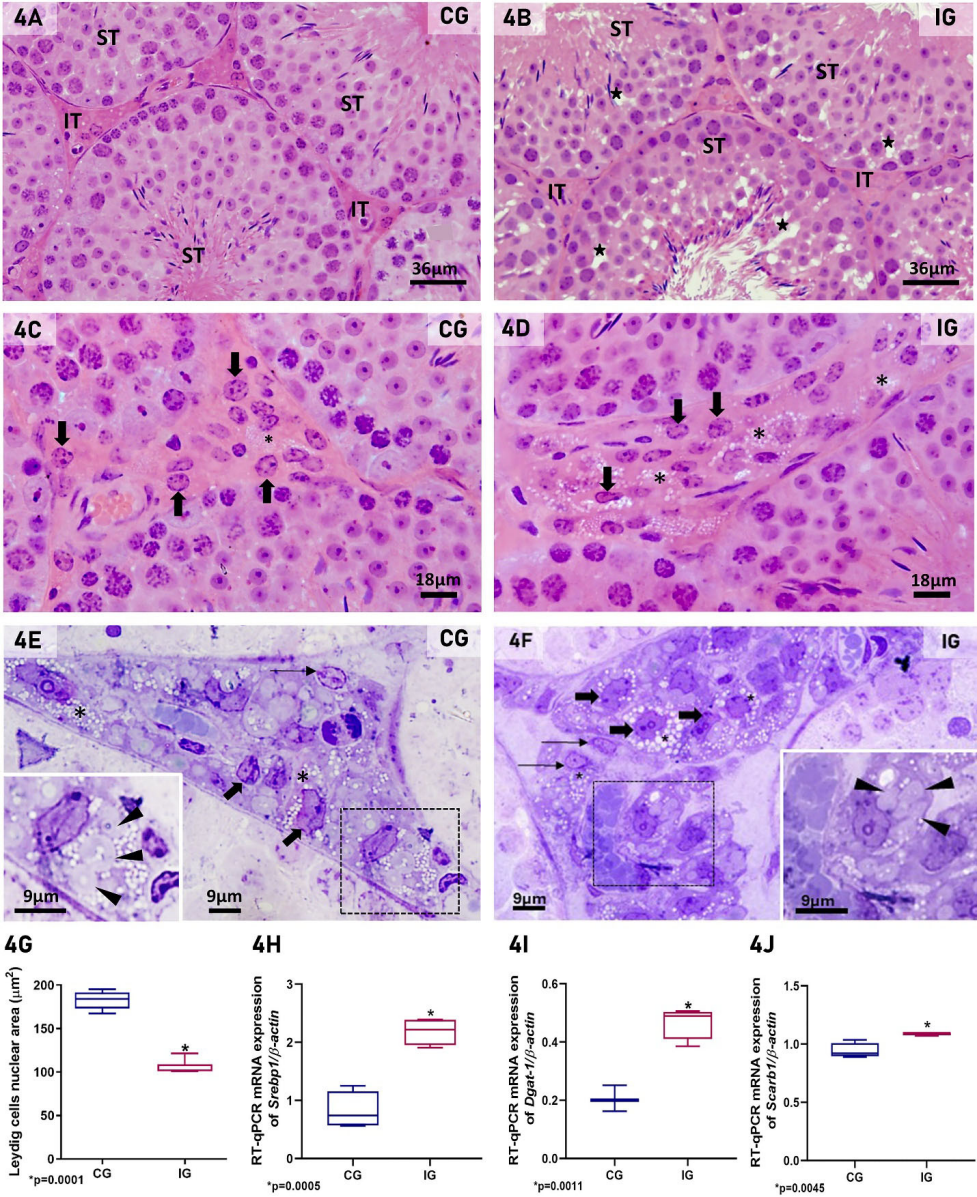
**(A–F)** Photomicrographs of testicular sections of animals from CG and IG stained with H.E. **(A-D)**, and semithin sections stained with toluidine blue **(E, F)**. In **(A)**, normal histoarchitecture of the seminiferous tubules (ST) and interstitial tissue (IT) is observed in CG. However, in **(B)** (IG), intraepithelial spaces are seen in the seminiferous tubules (ST; stars). IT, interstitial tissue. In **(C)**, the interstitial tissue exhibits typical LCs with round/ovoid nucleus (thick arrows) whereas in **(D)**, these cells show irregular and reduced nucleus (thick arrows), and numerous lipid inclusions (asterisks) in comparison to CG. In **(E, F)**, semithin sections show interstitial tissue containing macrophages (thin arrows) and LCs (thick arrows). Numerous and large lipid inclusions (asterisks) are observed in the LCs of IG when compared to CG. Note the spirally arranged cisternae in these cells (insets; arrowheads). **(G)** Significant reduction of LC nuclear area is observed in IG in comparison to CG. **(H–J)** A significant increase in the mRNA expression of *Srebp1*, *Dgat-1* and *Scarb1* is observed in IG when compared to CG. *p value.

The original figure is illustrated below.

In the published article, there were errors in **Table 1**.

**Table 1:** Primary and secondary antibodies.

Antibody name	Dilution	Catalog number	Manufacturer	RRID
Mouse anti-ACE2 monoclonal antibody	1:50	sc-73668	Santa Cruz Biotechnology, USA	AB_2861379
Rabbit anti-SARS-CoV-2 Spike Protein S1 Recombinant monoclonal antibody	1:250	MA5-36247	Invitrogen by Thermo Fisher Scientific	AB_2890589
Rabbit anti-SARS-CoV-2 nucleocapsid protein monoclonal antibody	1:3000	ab271180	Abcam, Cambridge, UK	–
Rabbit anti-IL-1β polyclonal antibody	1:400	ab9722	Abcam, Cambridge, UK	AB_308765
Goat anti-IL-6 (M19) polyclonal antibody	1:400	sc-1265	Santa Cruz Biotechnology, USA	AB_2127470
Mouse anti-TNF-α monoclonal [52B83] antibody	1:200	ab1793	Abcam, Cambridge, UK	AB_302615
Rabbit anti-CD163 monoclonal antibody	1:100	ab182422	Abcam, Cambridge, UK	AB_2753196
Rabbit anti-CD68 polyclonal antibody	1:100	ab125212	Abcam, Cambridge, UK	AB_10975465
Rabbit anti-17β-HSD6 polyclonal antibody	1:500	sc-393936	Santa Cruz Biotechnology, USA	AB_2891064
Rabbit anti-StAR polyclonal antibody	1:1000	PAS-95765	Invitrogen by Thermo Fisher Scientific	–
Mouse anti-testosterone polyclonal antibody	1:100	ab217912	Abcam, Cambridge, UK	AB_308765
Rabbit anti-IL-10 polyclonal antibody	1:500	sc-8438	Santa Cruz Biotechnology, USA	AB_627793
Rabbit anti-MIF polyclonal antibody	1:200	251415	ABBIOTEC, USA	AB_10636927
Rabbit anti-β-tubulin monoclonal antibody	1:8000	ab108342	Abcam, Cambridge, UK	AB_10866289
HRP-conjugated anti-rabbit secondary antibody	1:9000	A9169	Sigma-Aldrich, USA	
Alexa Fluor^®^488 anti-mouse antibody	1:1000	ab150113	Abcam, Cambridge, UK	AB_2576208
Alexa Fluor^®^488 anti-rabbit antibody	1:1000	ab150077	Abcam, Cambridge, UK	AB_2630356
Alexa Fluor^®^594 anti-mouse antibody	1:1000	ab150116	Abcam, Cambridge, UK	AB_2650601
Alexa Fluor^®^ 647 anti-rabbit antibody	1:1000	ab150075	Abcam, Cambridge, UK	AB_2752244
Alexa Fluor^®^647 anti-goat antibody	1:1000	ab150135	Abcam, Cambridge, UK	AB_2687955

The antibody “Donkey anti IL-6 (M19) polyclonal antibody” is incorrect. The actual antibody used was “Goat anti-IL-6 (M19) polyclonal antibody”. The “mouse anti-17beta-HSD7 antibody; 1:500 sc-393936; Santa Cruz Biotechnology, USA AB_2891064” is incorrect. The actual antibody used was the “Rabbit anti-17beta-HSD6; 2μg/ml; sc-101878; Santa Cruz Biotechnology, USA”. Regarding Alexa Fluor^®^ 488 anti-mouse antibody, the “catalog number (A11001), manufacturer (Molecular Probes by Life Technologies) and RRID” are incorrect. The correct is “catalog number ab150113; manufacturer (Abcam, Cambridge, UK) and RRID (AB_2576208)”. The data regarding the “Alexa Fluor^®^ 594 anti-rabbit antibody” are incorrect. The correct is “Alexa Fluor^®^ 647 anti-rabbit antibody; dilution 1:1000, catalog number (ab150075); manufacturer (Abcam, Cambridge, UK); RRID (AB_2752244)”. The “Alexa Fluor^®^ 647 anti-donkey polyclonal antibody” is incorrect. The correct is “Alexa Fluor^®^ 647 anti-goat antibody; dilution 1:1000; RRID (AB_2687955)”. The “Alexa Fluor^®^ 488 anti-rabbit antibody, Alexa Fluor^®^ 594 anti-mouse antibody, and their respective dilution, catalog number, manufacturer and RRID” were missing and were added to **Table 1**. The corrected **Table 1** is below.

**Methods** (items *2.6* - page 4 and *2.7* -page 5), where it reads “17beta-HSD7”, the correct word is “17beta-HSD6”; this was incorrectly described by mistake.

A correction has been made to the sections:

**2. Material and methods**, *2. 6 Immunohistochemistry and immunofluorescence analyses*, Paragraph 2, Line 9: “anti-17β-HSD6 polyclonal antibody”

*2.7 Double immunofluorescence analysis*, Lines 4-11:

“Moreover, to confirm if the LCs express an inflammatory profile, double immunofluorescences for detection of 17β-HSD6+IL-6, 17β-HSD6+IL-1β and StAR+TNF-α were also performed. The double immunofluorescence reactions were performed according to de Santi et al. (2022). After antigen recovery, the sections were incubated overnight at 4°C with the following primary antibodies (**Table 1**): anti-human ACE2 monoclonal antibody, anti-17β-HSD6 polyclonal antibody or anti-StAR polyclonal antibody.”

Legend: Figure 1 (J, K): where it reads “17beta-HSD7”, the correct word is “17beta-HSD6”.

The original version of this article has been updated.

