# Correction: SARS-CoV-2 exploits steroidogenic machinery, triggers lipid metabolism for viral replication and induces immune response in Leydig cells of K18-hACE2 mice

**DOI:** 10.3389/fcimb.2026.1871852

**Published:** 2026-05-29

**Authors:** Salmo Azambuja de Oliveira, André Acácio Souza da Silva, Barry T. Hinton, Giovanni Freitas Gomes, Thiago Mattar Cunha, Paulo Sérgio Cerri, Estela Sasso-Cerri

**Affiliations:** 1 Department of Morphology and Genetics, Federal University of São Paulo, São Paulo, SP, Brazil; 2 Department of Cell Biology, School of Medicine, Virginia University, Charlottesville, VA, United States; 3 Center for Research in Inflammatory Diseases, Faculty of Medicine of Ribeirão Preto - USP, Ribeirão Preto, SP, Brazil; 4 Laboratory of Histology and Embryology, Department of Morphology, Genetics, Orthodontics and Pediatric Dentistry, School of Dentistry, São Paulo State University (UNESP), Araraquara, SP, Brazil

**Keywords:** COVID-19, testosterone, cholesterol, lipogenesis, nucleocapsid, cytokines, macrophages, electron microscopy

In the published article, information was missing regarding the ethical documentation and source of the human tissue sample used in **Figure 1F**.

A correction has been made to the **Ethics Statement** section:

“This study utilizes a human testicular tissue photomicrograph (**Figure 1**) obtained from a histological slide provided by IPC Medicina Diagnóstica (Araraquara, SP, Brazil). Ethics committee approval was not required according to local legislation (Brazilian National Health Council Resolution CNS 466/2012) because the material consists solely of a de-identified (anonymous) archival sample, with no possibility of identifying the donor”.

The original version of this article has been updated.

